# Congenital myasthenic syndrome due to a *TOR1AIP1* mutation: a new disease pathway for impaired synaptic transmission

**DOI:** 10.1093/braincomms/fcaa174

**Published:** 2020-10-18

**Authors:** Judith Cossins, Richard Webster, Susan Maxwell, Pedro M Rodríguez Cruz, Ravi Knight, John Gareth Llewelyn, Ji-Yeon Shin, Jacqueline Palace, David Beeson

**Affiliations:** Neurosciences Group, Weatherall Institute of Molecular Medicine, John Radcliffe Hospital, University of Oxford, Oxford OX3 9DS, UK; Neurosciences Group, Weatherall Institute of Molecular Medicine, John Radcliffe Hospital, University of Oxford, Oxford OX3 9DS, UK; Neurosciences Group, Weatherall Institute of Molecular Medicine, John Radcliffe Hospital, University of Oxford, Oxford OX3 9DS, UK; Neurosciences Group, Weatherall Institute of Molecular Medicine, John Radcliffe Hospital, University of Oxford, Oxford OX3 9DS, UK; Department of Clinical Neurology, John Radcliffe Hospital, Oxford OX3 9DU, UK; Department of Clinical Neurology, John Radcliffe Hospital, Oxford OX3 9DU, UK; Neurology Department, University Hospital of Wales, Heath Park, Cardiff CF14 4XW, UK; Department of Medicine, Columbia University Medical Centre, New York, NY 10032, USA; Department of Clinical Neurology, John Radcliffe Hospital, Oxford OX3 9DU, UK; Neurosciences Group, Weatherall Institute of Molecular Medicine, John Radcliffe Hospital, University of Oxford, Oxford OX3 9DS, UK

**Keywords:** neuromuscular junction, congenital myasthenic syndromes, nuclear envelope protein, myasthenia, envelopathy

## Abstract

Congenital myasthenic syndromes are inherited disorders characterized by fatiguable muscle weakness resulting from impaired signal transmission at the neuromuscular junction. Causative mutations have been identified in genes that can affect the synaptic function or structure. We identified a homozygous frameshift deletion c.127delC, p. Pro43fs in *TOR1AIP1* in two siblings with limb-girdle weakness and impaired transmission at the neuromuscular synapse. *TOR1AIP1* encodes the inner nuclear membrane protein lamin-associated protein 1. On muscle biopsy from the index case, lamin-associated protein 1 was absent from myonuclei. A mouse model with lamin-associated protein 1 conditionally knocked out in striated muscle was used to analyse the role of lamin-associated protein 1 in synaptic dysfunction. Model mice develop fatiguable muscle weakness as demonstrated by using an inverted screen hang test. Electromyography on the mice revealed a decrement on repetitive nerve stimulation. *Ex vivo* analysis of hemi-*diaphragm* preparations showed both miniature and evoked end-plate potential half-widths were prolonged which was associated with upregulation of the foetal acetylcholine receptor γ subunit. Neuromuscular junctions on *extensor digitorum longus* muscles were enlarged and fragmented, and the number of subsynaptic nuclei was significantly increased. Following these findings, electromyography was performed on cases of other nuclear envelopathies caused by mutations in LaminA/C or emerin, but decrement on repetitive nerve stimulation or other indications of defective neuromuscular transmission were not seen. Thus, this report highlights the first nuclear membrane protein in which defective function can lead to impaired synaptic transmission.

## Teaser

A mutation in *TOR1AIP1* causes a new type of congenital myasthenic syndrome, characterized by limb girdle fatiguable muscle weakness and compound muscle action potential decrement on repetitive nerve stimulation. A mouse knockout model shows impaired neurotransmission, fragmented neuromuscular junctions, increased synaptic nuclei and up-regulation of foetal acetylcholine receptors.

## Graphical Abstract

**Graphical Abstract:**
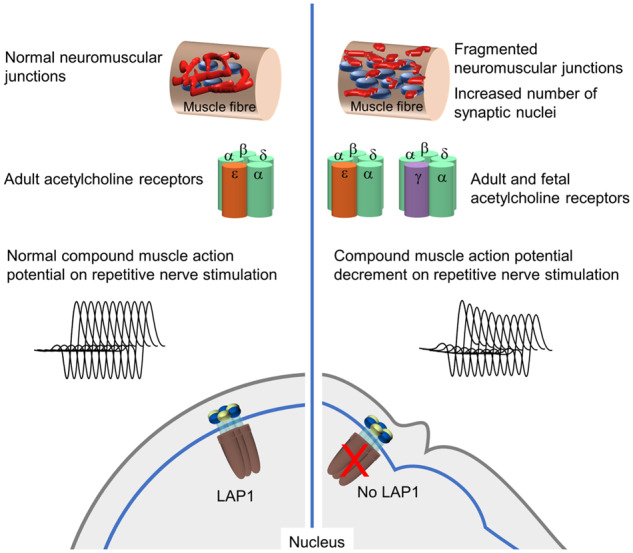

## Introduction

Congenital myasthenic syndromes (CMS) comprise a heterogeneous group of rare inherited disorders characterized by fatiguable muscle weakness (Rodriguez Cruz *et al.*, 2018) that results from impaired signalling at the neuromuscular junction (NMJ). Inheritance is usually autosomal recessive. The majority of mutations that underlie CMS are found in genes encoding proteins with expression largely restricted to this synapse, for example, muscle nicotinic acetylcholine receptor (AChR) subunits, *RAPSN*, *MUSK*, *COLQ* and *DOK7* (Finlayson *et al.*, 2013). More recently, the use of next generation sequencing has helped to expand the repertoire of CMS-associated genes to include many proteins involved in presynaptic cholinergic transmission and additional genes encoding proteins that are ubiquitously expressed. These include *DPAGT1*, *ALG2*, *ALG14* and *GMBBP* that are involved in N-linked and O-linked glycosylation (Belaya *et al.*, 2012; Cossins *et al.*, 2013; Belaya *et al.*, 2015; Rodriguez Cruz *et al.*, 2016; Luo *et al.*, 2017). The age of onset, pattern of weakness and response to therapy can vary significantly depending on the pathogenic mechanism of each mutation (Rodriguez Cruz *et al.*, 2014).

Mutations in genes coding for proteins in the nuclear membrane were first highlighted by the finding that X-linked Emery–Dreifuss Muscular Dystrophy was due to defective emerin (*EMD* gene) located in the inner nuclear membrane (Bione *et al.*, 1994; Nagano *et al.*, 1996). Subsequently, proteins located in the outer nuclear membrane, which is morphologically continuous with the endoplasmic reticulum, the inner nuclear membrane, or on the inner side of the inner nuclear membrane were found to underlie a series of disorders collectively known as nuclear envelopathies. The defects in the integrity of the nuclear membrane are thought to compromise communication between the nucleus and the cytoplasm, chromatin stability and transcriptional control. Although ubiquitously expressed, defective nuclear envelope proteins give rise to disorders that are remarkably tissue-specific. Skeletal and cardiac muscle are frequently targeted, as illustrated by Emery–Dreifuss Muscular Dystrophy that manifests as slowly progressive muscle wasting, particularly of humeroperoneal regions, joint contractures and cardiac anomalies. Lamin-associated protein 1 (LAP1) is ubiquitously expressed, spans the inner nuclear membrane and is encoded by the gene *TOR1AIP1* (Kondo *et al.*, 2002). The N-terminal domain of LAP1 interacts with A-type lamins and emerin within the nucleoplasm (Senior and Gerace, 1988; Shin *et al.*, 2013). The C-terminal luminal domain interacts with and is thought to activate TorsinA, an AAA+ATPase which resides in the lumen of the nuclear envelope (Zhao *et al.*, 2013). The LAP1 luminal domain is highly homologous with the C-terminal domain of LULL1, which also interacts with TorsinA within the lumen of the endoplasmic reticulum (Zhao *et al.*, 2013). Mutations in proteins that interact with LAP1 can give rise to a range of muscular dystrophies (Worman *et al.*, 2010; Worman and Dauer, 2014; Muchir and Worman, 2019). Rare recessive mutations in *TOR1AIP1* have been reported to cause limb-girdle muscular dystrophy or dystonia, with cardiomyopathy or a severe multisystem disorder (Dorboz *et al.*, 2014; Kayman-Kurekci *et al.*, 2014; Ghaoui *et al.*, 2016). Here, we characterize a novel disease mechanism for congenital myasthenic syndrome in which a mutation in a nuclear envelope protein, LAP1, gives rise to impaired neuromuscular transmission which has not been seen in other associated nuclear envelopathies, but highlights a potential novel pathway for the disruption of synaptic transmission.

## Materials and methods

### Ethical statement

Written informed consent was obtained according to the Declaration of Helsinki from the patients for DNA analysis, biopsy analysis and publication and is available for review. Ethical approval was obtained from OXREC B: 04.OXB.017 and Oxfordshire REC C 09/H0606/74.

### Next generation sequencing

Genomic DNA from the index case was subject to whole exome sequencing (Welcome Trust Centre for Human Genetics, Oxford, UK) funded by Oxford NIHR Biomedical Research Centre Genomic Medicine Theme. Exome library was captured from 3 µg of genomic DNA using Roche Nimblegen SeqCap EZ Human Exome Library v2.0. The libraries were sequenced by 100 nt paired-end reads on the Illumina HiSeq platform. The obtained sequences were mapped to the human genome build hg19 by using Novoalign software (Novocraft Technologies). Variants were called using the Samtools program. We used ANNOVAR software to annotate and separate non-synonymous substitutions, splicing mutations and mutations in 3' or 5' UTRs.

### Immunofluorescence staining of human muscle biopsy

Muscle biopsies were frozen in liquid nitrogen-cooled isopentane and stored at −80°C. Ten micrometre sections were cut using a Leica CM1900 cryostat at −16°C. Tissue sections were dried and incubated with a blocking buffer (PBS containing 5% foetal calf serum). Endplates were visualized by incubation with 2 µg/ml 594-alexafluor-tagged α-Bungarotoxin (594-α-BuTx; Invitrogen) 1 h. Sections were washed and incubated with 6.7 µg/ml 488-alexafluor-tagged Fasciculin-2 (488-Fasciculin-2; custom made, Invitrogen). Atlas rabbit anti-TOR1AIP1 primary antibody HPA050546 (Cambridge Bioscience, UK) was used at 0.6 µg/ml and secondary Alexa Fluor tagged antibodies (Invitrogen) were used at 1 µg/ml. All staining was carried out in a blocking buffer.

### Isolation and culture of human muscle cells

Human myoblasts were disassociated from a muscle biopsy using 0.05% trypsin and 0.02% EDTA in Hanks balanced solution and were cultured in Skeletal Muscle Cell Growth Medium (ECACC). After a week in culture, myoblasts were enriched by pulling down with an anti-CD56 monoclonal antibody (AbD Serotec). Cells were differentiated into myotubes by culturing in DMEM containing 2% foetal calf serum.

### Western blotting

Cells were harvested from culture plates using trypsin and were lysed by rotating for 1 h at 4 °C in lysis buffer (50 mM Tris-HCl pH 7.5, 1% triton X-100 and 500 mM NaCl) supplemented with protease inhibitor cocktail (Sigma, P8340). Cell debris was removed by centrifugation and the protein concentration was assessed using a Pierce BCA protein assay kit (Thermo Fisher Scientific). 20 µg was subject to SDS PAGE and transferred to nitrocellulose. LAP1 was detected using 1.5 µg/ml Atlas anti-TOR1AIP1 antibody HPA050546 (Cambridge Bioscience) and housekeeping protein α-tubulin was detected using monoclonal antibody ascites diluted 1:4000 (Sigma T5168). Secondary HRP-conjugated antibodies were from Dako and were visualized using ECL (Sigma).

### Model mice

All procedures were performed in accordance with the Animals (Scientific Procedures) Act 1986 and the Home of code of practice and were approved by the University Ethical Review Panel. Animals were housed at 19–23°C, humidity 45–64% with 12 h light/12 h of dark, in individual ventilated caging (IVC) with aspen chip bedding (Datesand Eco4). They were fed with SDS RM1 food, water was RO chlorinated, and sizzle nest and tubes were included.

Mice with floxed *Tor1aip1* (Shin *et al.*, 2013) were crossed with B6.FVB(129S4)-Tg(Ckmm-cre)5Khn/J stock number 006475 (Mck-Cre) mice from The Jackson Laboratory, in order to conditionally knock out *Tor1aip1* in striated muscle (referred to as M-LAP1^−/−^ mouse in this article). In this mouse line, Cre is expressed under the muscle creatine kinase (CK) promoter, which is active from day E13 in the developing embryo and is maintained throughout adulthood (Lyons *et al.*, 1991). Muscle strength and fatiguability of six M-LAP1^−/−^ mice and six littermate controls was assessed using an inverted screen hang test as previously described (Webster *et al.*, 2013; Vanhaesebrouck *et al.*, 2019) and illustrated in Supplementary Fig. 2a.

### Electromyography in M-LAP1^−^^/^^−^ mice

Repetitive nerve stimulation of three M-LAP1^−/−^ mice and three littermate controls at 6, 9 and 12 weeks of age was performed as described previously (Webster *et al.*, 2013). Detailed methods for EMG and for endplate potential recordings and two-electrode voltage-clamp of endplate current recordings in phrenic nerve-*diaphragm* preparations are described in Supplementary material.

### Immunofluorescence staining of endplate regions from mouse muscle

The method for whole teased fibre staining of *extensor digitorum longus* (EDL), *soleus* (SOL) and *diaphragm* was based on a previously published method (Vanhaesebrouck *et al.*, 2019) and is given in detail in Supplementary material. For nuclei counting, AChRs were stained with 594-α-BuTx as described above and then individual fibres were teased apart. Fibres were mounted in Confocal Matrix containing DAPI onto microscopy slides, which were then coded so that the image acquisition and analysis were carried out blinded. Stained muscles were visualized using an Olympus IX71 wide-field fluorescence microscope. Images were captured using Simple PCI (Digital Pixel) and analysed using ImageJ. Number of mice used for each analysis was as described in the figure legends.

### Quantification of endplate AChR

Quantification of AChR for three M-LAP1^−/−^ mice and three littermate controls aged 12 weeks was based on the method described (Schwarz *et al.*, 2000). Details are in Supplementary material. The endplate-specific ^125^I-α-BuTx binding was expressed as cpm/mm^2^ of muscle fibres, allowing comparisons between different diaphragms.

### RNA isolation, cDNA synthesis and quantitative PCR

At 6 and 12 weeks of age three control and three model mice were sacrificed, and EDL muscles were dissected, snap frozen in liquid nitrogen-cooled isopentane, and stored at −80°C. Total RNA was extracted using TRIzol^TM^ Reagent (ThermoFisher Scientific) according to the manufacturer’s instructions. Contaminating genomic DNA was removed using the TURBO DNA-free^TM^ kit (ThermoFisher Scientific). Quality and concentration was assessed using a NanoDrop^TM^ (ThermoFisher Scientific) and Agilent Tapestation. RNA-seq was carried out by Novogene. See Supplementary material for analysis methodology. cDNA was generated from 2 µg RNA using a High-Capacity RNA-to-cDNA Kit (Life Technologies Ltd). qPCR was carried out on a QuantStudio7 (Life Technologies Ltd) using TaqMan Gene Expression Master Mix and Taqman Gene Expression Assay kits (Life Technologies Ltd). A list of the genes and probe IDs is given in Supplementary Table 1. The internal gene control was *Hprt1*. Analysis methods are described in Supplementary material.

### Statistical analysis

Unpaired two-tailed *t*-tests were used to analyse electrophysiology data and AChR expression on hemi-*diaphragm* preparations using Graphpad Prism. ANOVA analysis with Tukey’s multiple comparison was used to analyse NMJ morphological data and subsynaptic nuclei numbers. For analysis of data from qPCR, one-tailed unpaired Welch’s *t*-test analysis was performed to analyse differences in target gene expression between control and M-LAP1^−/−^ mice.

### Data availability

Data are available from the authors upon reasonable request.

## Results

### Clinical features

**Figure 1:**
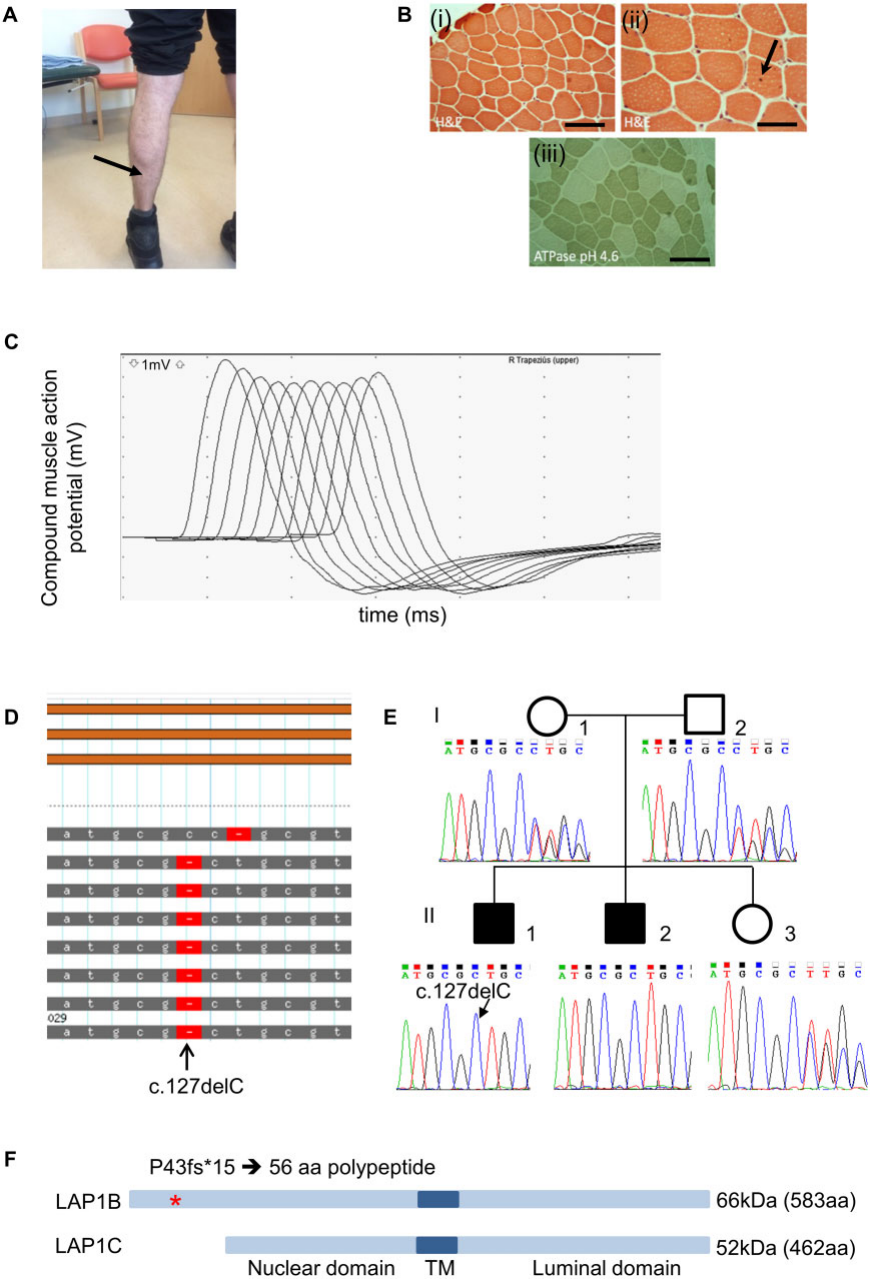
**Clinical, histological and genetic analysis of cases 1 and 2.** (**A**) Mild muscle wasting (arrow) in *medial gastrocnemius* is shown in individual 1. (**B**) H&E staining of *quadriceps* muscle biopsy from individual 1 (when he was 9 years old) shows mild variation in fibre size (i) and an occasional central nucleus indicated by arrow (ii). ATPase pH 4.3 indicates a predominance of type I muscle fibres (iii). Scale bar in (i) and (iii) = 100 µm and in (ii) is 50 µm. (**C**) Example RNS trace from affected individual 1 from the *trapezius* showing the characteristic envelope or U shape that is typical of decrement recording from a NMJ with a postsynaptic myasthenic disorder. Muscle was stimulated with a train of 10 stimuli at 3 Hz and CMAPs recorded. The traces from each stimulus are overlaid. Decrement is recorded at the fourth stimulus. (**D**) Exome sequencing of genomic DNA from individual 1 showing homozygous deletion in exon 1 at position c.127delC (p.P43fs). (**E**) Sanger sequencing of exon 1 showing segregation of c.127delC within the family. (**F**) Schematic diagram showing location of mutation (*) within the protein. Mutation is predicted to truncate LAP1B whilst leaving LAP1C unaffected. TM = transmembrane domain.

The proband, Individual 1, from unrelated parents, was born at term following an uneventful pregnancy with preserved foetal movements. All early developmental milestones were normal. the presentation was at age 6 years with walking difficulties and fatigue. He struggled with running and walking upstairs and had unexplained recurrent falls. Cognitive function and academic performance were normal. Examination at age 10 showed moderate proximal muscle weakness in the limbs and diminished muscle bulk around the shoulder girdle with mild wasting of *deltoid* muscle. Eye movements were full and no weakness was found in facial muscles. Mild distal contractures were present at the ankles. The severity of symptoms has remained stable over the years with perhaps some improvement from age 11 to 16 and no marked progression up to the present time. Currently, at age 26, he complains of being unable to put on muscle bulk despite going to the gym regularly, reduced exercise tolerance and difficulties lifting weights with his upper limbs. A recent examination showed no ptosis, preserved eye movements, full power in facial and neck muscles, but generalized reduced muscle bulk with mild wasting of the *deltoid* and *medial gastrocnemius* muscles (Fig. 1A). Of note, he was unable to lift his arms at 90° for more than 45 s (normal > 240 s). More detailed clinical information is in Table 1.

**Table 1:** Clinical features of individuals homozygous for the c.127delC p. P43fs* mutation in *TOR1AIP1*

	Individual 1	Individual 2
Gender	M	M
Current age	31	33
Pregnancy	Normal	Normal
Age at onset	6 years	Retrospective report of fatigue in childhood
Presenting symptoms	Walking difficulties, fatigue on exercise, recurrent falls	Reduced exercise tolerance, mild proximal weakness, fatigue on exercise
Ptosis	No	No
Ophthalmoparesis	No	No
Facial weakness	No	No
Bulbar weakness	No	No
*Deltoid*	MRC 4	MRC 4+
*Supra* and *infraspinatus*	MRC 4	MRC 4
*Brachioradialis*	MRC 4	MRC 4
*Extensor digitorum communis*	MRC 4+	MRC 4+
*Abductor pollicis brevis*	MRC 4+	MRC 5
Hip extensors	MRC 5−	MRC 5
Hip abductors	MRC 5−	MRC 5
Knee flexors	MRC 5	MRC 5−
Ankle dorsiflexion	MRC 4	MRC 4
Muscle wasting	*deltoid*, *medial gastrocnemius*	Shoulder girdle, *medial gastrocnemius*
Ankle contractures	Mild	Mild
QMG	9/39	8/39
QMG following treatment (pyridostigmine)	7/39	6/39

QMG = quantitative myasthenia gravis score; MRC = Medical Research Council score for muscle weakness.

Investigations included a *quadriceps* muscle biopsy at age 9 years (Fig. 1B) that showed mild variation in fibre size and only occasional internal nuclei. Type I fibre predominance was evident from the ATPase pH 4.3 staining, but there an absence of fibre type grouping (the presence of which would indicate denervation). Muscle MRI at age 11 years was reported as normal. Neurophysiological studies at age 26 showed significant decrement on repetitive nerve stimulation at 3 Hz from *anconeus* muscle of more than 40%, and 21% from *trapezius* (example trace shown in Fig. 1c, and data for right *trapezius* are shown in Supplementary Table 2). Additional testing on *abductor digiti minimi* and *abductor pollicis brevis* muscles showed a 12% decrement that partially alleviated following a period of maximum voluntary contraction. Single fibre EMG examination of *extensor digitorum communis* showed 36% increased jitter and 64% blocking from a total of 11 pairs evaluated. The concentric needle examination of *deltoid* showed a full interference pattern with a moderate excess of irregular or polyphasic motor unit potentials. Nerve conduction studies were normal. A cardiac assessment including ECG, echocardiogram and 24-h Holter monitoring was normal. He tested negative for AChR, muscle-specific tyrosine kinase (MuSK) and voltage-gated calcium channel antibodies by radioimmuno assay and also for clustered-AChR (Leite *et al.*, 2008), MuSK and low-density lipoprotein receptor-related protein 4 (LRP4) antibodies by cell-based assays. The CK levels in serum were modestly raised at 401 UI/l. The specific reference range for CK in serum depends on individual laboratories but values greater than 200 are in general considered abnormal. Pharmacological treatment with acetylcholinesterase inhibitor pyridostigmine 90 mg three times daily resulted in symptomatic improvement in the form of increased exercise tolerance. This was corroborated objectively with an improvement in his quantitative myasthenia gravis test (QMG) from 9/39 to 7/39. The subsequent addition of salbutamol up to 6 mg twice daily did not give any further improvement.

The elder brother of the proband was born at term following a normal pregnancy. Developmental milestones were normal and no concerns were raised during infancy and childhood although he recalls being slower and getting tired more quickly than peers at school. He was assessed at 30 years of age following his brother’s diagnosis and analysis of his DNA. He reported reduced exercise tolerance and was found to have mild proximal weakness and fatigue affecting upper limb function and neck. Examination showed mild wasting of shoulder girdle muscles and *medial gastrocnemius* (clinical details in Table 1). Of note, he was unable to lift his arms at 90° for more than 75 s (normal > 240 s). Investigations showed mildly elevated serum CK levels at 229 UI/l. Neurophysiological studies showed a marked decrement of 21% on repetitive nerve stimulation for *trapezius* muscle, but no decrement was observed for *abductor digiti minimi*. Single fibre EMG examination of *extensor digitorum communis* showed increased jitter and block in 16 out of 25 pairs, and single fibre EMG of *deltoid* showed a markedly increased jitter and block in 9 out of 11 pairs evaluated. Cardiac evaluation was normal. Treatment with pyridostigmine resulted in symptomatic improvement of his upper limb function and neck symptoms. His QMG score improved from 8/39 to 6/39.

### Genetic analysis

Next generation sequencing (whole exome sequencing) of the proband’s genomic DNA identified a homozygous single nucleotide deletion, c.127delC p. Pro43fs (Ensembl transcript ENST00000606911.6), in exon 1 of *TOR1AIP1* (Fig. 1D), which encodes LAP1. Sanger sequencing of family members showed that the proband’s brother had the same homozygous variant. Segregation of the variant within the family (Fig. 1E), and the low MAF of 1.14e−05, is consistent with an autosomal recessive disorder. As illustrated in Fig. 1F, two human isoforms of LAP1 arise from the use of different methionine initiation codons: LAP1B (583 amino acids) and LAP1C (462 amino acids). Variant c.127delC, (p.Pro43fs) is predicted to truncate LAP1B to produce a protein of only 58 amino acids, while leaving LAP1C unaffected.

### *TOR1AIP1* mutation c.127delC abrogates expression of LAP1B and causes morphological changes to myonuclei in the patient muscle biopsy

**Figure 2:**
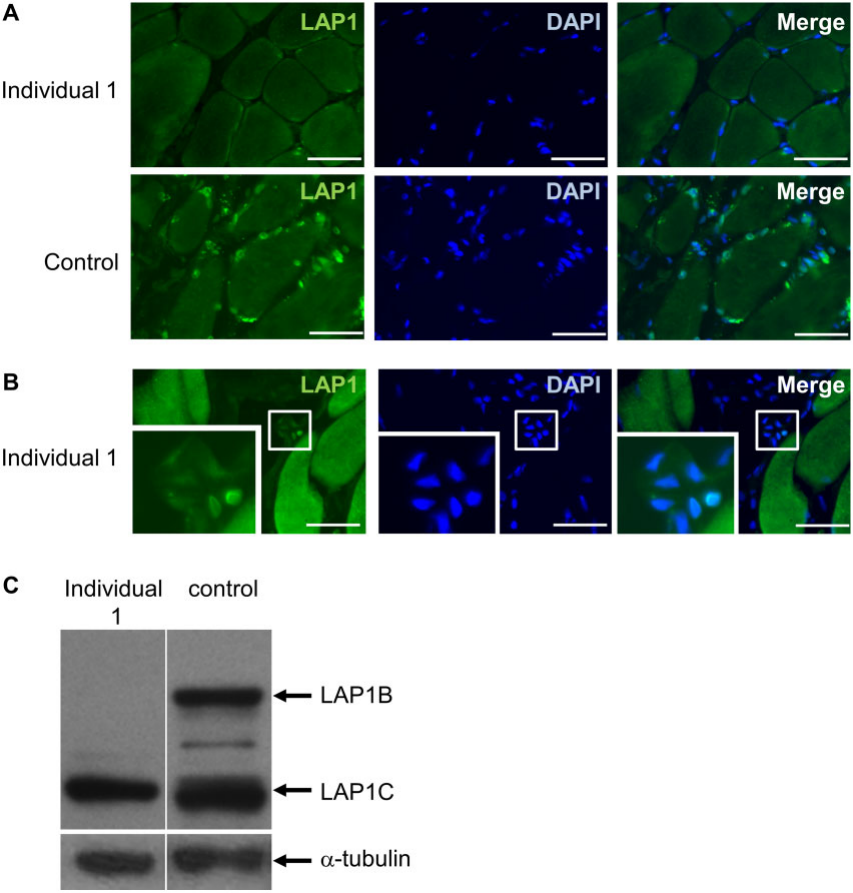
**Analysis of LAP1 expression in muscle biopsy from *trapezius* and muscle cells from individual 1.** (**A**) LAP1 is expressed in myonuclei in a control muscle biopsy but is undetectable in a myonuclei in a muscle biopsy from *trapezius* muscle from individual 1. Scale bar = 50 µm. (**B**) LAP1 can be detected in nuclei within endothelial cells in the patient biopsy. Inset shows the selected region of interest at higher magnification. Scale bar = 50 µm. (**C**) Western blot showing expression of LAP1B and LAP1C in muscle cells cultured in differentiation medium. Both isoforms are expressed in control cells but LAP1B is absent in muscle cells from individual 1.

A small section of discarded *trapezius* muscle was obtained from the proband, aged 26, while undergoing a separate procedure. Immunofluorescence staining of the muscle showed no detectable expression of LAP1 in myonuclei compared with a control muscle biopsy (Fig. 2A). The antibody used here can recognize both LAP1B and LAP1C, and so this supports previous findings that LAP1B is the major isoform expressed in myonuclei (Kayman-Kurekci *et al.*, 2014). Faint staining, representing expression of LAP1C, was observed in blood vessel endothelial cell nuclei (Fig. 2B). To confirm a lack of the LAP1B isoform expression in our patient, we cultured muscle cells in differentiation medium and analysed protein expression by western blot. LAP1B expression is upregulated when control muscle cells are cultured in a differentiation medium, as shown in Fig. 2C and previously described (Shin *et al.*, 2017), but muscle cells from the proband do not express this isoform (Fig. 2c, and full size blots are shown in Supplementary Figure 1). LAP1C levels were similar in control and patient muscle cells. This was expected, because even though LAP1B is the major isoform in mature muscle, high levels of LAP1C expression in proliferating and differentiating cultured muscle cells has been previously observed (Shin *et al.*, 2017). Thus, from these results, we confirm the muscle isoform of LAP1, termed LAP1B, is ablated by the c.127delC mutation.

**Figure 3:**
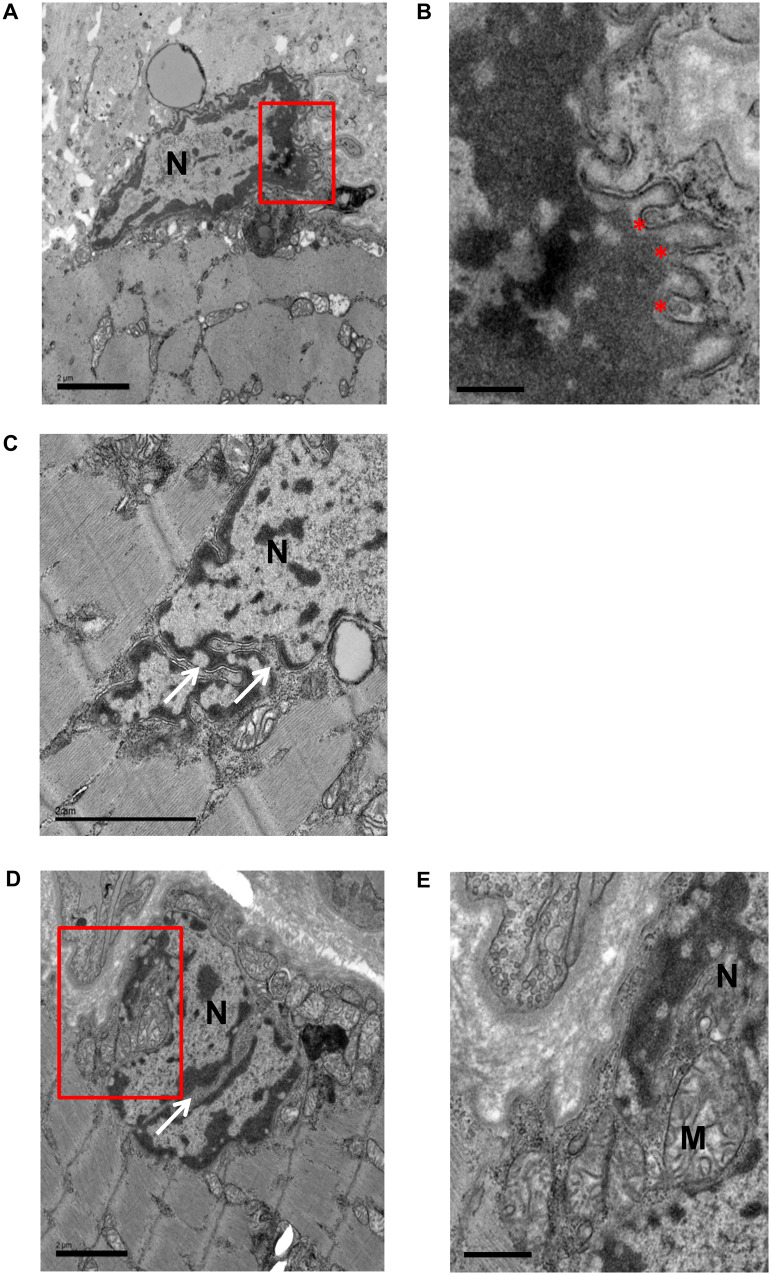
**Transmission electron micrographs showing abnormal myonuclei in patient biopsy.** The red regions of interest in **A** and **D** are shown enlarged in **B** and **E**, respectively. Convoluted nuclear envelope is visible in all nuclei shown. Other abnormalities include detached chromatin, where there are gaps between inner nuclear envelope and electron dense chromatin (**B**), nuclear envelope blebs (*) in **B**, and cytoplasmic channels (white arrows) in **C** and **D** with some infiltration of mitochondria (**E**). M = mitochondria; N = nucleus. Scale bars: 2 µm in (**A**), (**C**) and (**D**); 0.5 µm in (**B**); 1 µm in (**E**).

Because the patients had impaired neuromuscular transmission, we assessed if there was a loss of AChRs at the NMJ. Muscle cryo-sections were stained with 594-alexafluor-tagged α-Bungarotoxin (594-α-BuTx) to visualize AChRs and also with 488-Fasciculin-2 to visualize acetylcholinesterase. We only located two motor endplates, and in these the AChRs and acetylcholinesterase were both clearly expressed and co-localized (Supplementary Fig. 2). We were unable to find any endplates in the muscle from the patient using transmission electron microscopy and thus do not have data on the ultrastructure of patient NMJs. However, we were able to gain ultrastructural information about the myonuclei. Many abnormalities were observed, including convoluted nuclear periphery, heterochromatin which has detached from the inner nuclear membrane, and nuclear envelope blebs (Fig. 3A and enlarged in Fig. 3B). Other nuclear deformities included cytoplasmic channels with some infiltrating mitochondria (Fig. 3c–e).

### Conditional deletion of LAP1 from mouse muscle causes defective neurotransmission

To further analyse the post-synaptic role of LAP1 at NMJs, we used a mouse model in which *Tor1aip1* is conditionally knocked out in striated muscle (Shin *et al.*, 2013). This mouse model is referred to as M-LAP1^−/−^.

**Figure 4:**
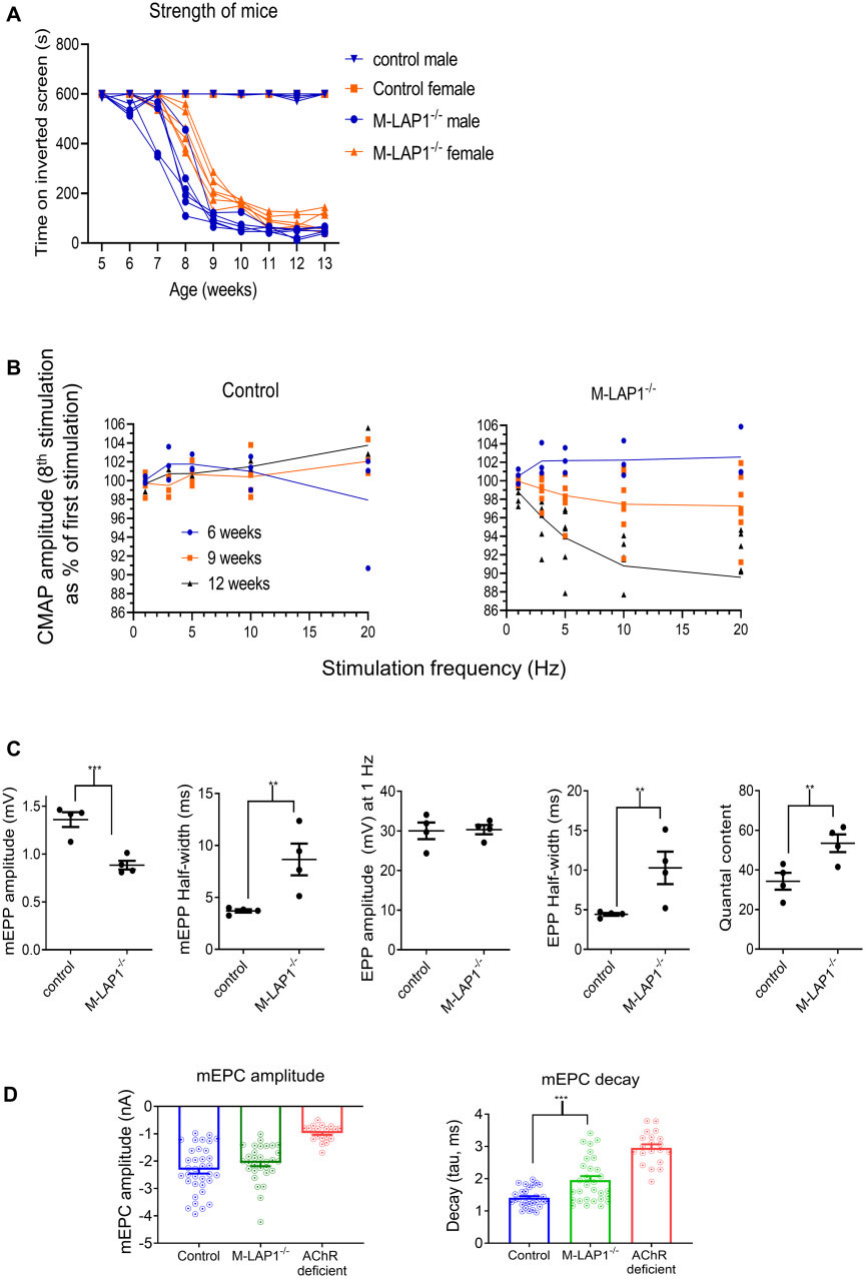
**Analysis of strength and electrophysiology of M-LAP1^−/−^ mice.** (**A**) Strength test of M-LAP1^−/−^ mice and littermate controls using an inverted screen hang test. The time the mice hung onto the mesh was measured, and the endpoint was 10 min. Six mice for each group were measured, error bars show standard deviation. (**B**) EMG data from *gastrocnemius* of M-LAP1^−/−^ and littermate control mice. Summary of EMG data showing how decrement (represented as the value of the 8th stimulus as a percentage of the first) varies with stimulation frequency for mice aged 6, 9, and 12 weeks (*n* = 3 mice control 6 and 12 weeks and M-LAP1^−/−^ 6 weeks; 4 mice control 9 weeks; 7 mice M-LAP1^−/−^ 9 and 12 weeks). (**C**) Single electrode analysis of muscle membrane potential in *diaphragm*/phrenic nerve preparations from 12-week-old mice. Summary of the data showing mEPP and EPP amplitudes, mEPP and EPP half widths and quantal content. (n = four mice per group; *P < 0.05, **P < 0.01, ***P < 0.001). (**D**) Measurements of endplate current using two-electrode voltage clamp potential in *diaphragm*/phrenic nerve preparations from 12-week-old mice. Summary of decay (tau) and mEPC amplitude. Also included is data from an AChR deficiency mouse model in which only foetal AChRs are expressed, for comparison. (*n* = three mice per group; ***P < 0.001).

First, we analysed the fatiguability of muscle strength over time, to establish the age at which they start to show weakness. For this we used an inverted screen test (Supplementary Fig. 3a), which we have found previously provides an effective measure of myasthenic weakness (Webster *et al.*, 2013; Vanhaesebrouck *et al.*, 2019). Loss of LAP1 in mouse striated muscle caused the mice to lose strength from around 6 weeks of age (Fig. 4A) which coincided with the age at which mice stop gaining weight (Supplementary Fig. 3b). After a gradual loss of strength from age 6 to 10 weeks, the mice maintained an abnormally low but stable level of strength for a further 4 weeks.

Electromyography was performed to demonstrate that this fatiguable weakness was due to a functional defect in neuromuscular transmission, which develops in the mature mice. At 6 weeks, before the mice show overt weakness, there was no compound muscle action potential (CMAP) decrement in either controls or M-LAP1^−/−^ mice. At 9 weeks of age, when weakness is developing, there was a small degree of CMAP decrement which is exacerbated by higher frequency stimulation (∼3% decrement at 20 Hz). By 12 weeks of age, when the M-LAP1^−/−^ mice were stably weak, the degree of CMAP decrement had increased to ∼11% at 20 Hz. In control mice, there was no CMAP decrement at any time point. An example trace showing CMAP decrement is shown in Supplementary Fig. 4a, and the data are summarized in Fig. 4b and Supplementary Fig. 4b. Thus, we demonstrate a functional defect in neurotransmission at the neuromuscular synapse in M-LAP1^−/−^ mice that develops after 6 weeks of age and coincides with the development of fatiguable muscle weakness.

The nature of this defect was further investigated in *diaphragm*/phrenic nerve isolated preparations from 12-week-old animals. Various parameters of neurotransmission were investigated with a single electrode analysis of muscle membrane potential. Example traces shown in Supplementary Fig. 4c demonstrate both miniature end-plate potential (mEPP) and end-plate potential (EPP) prolongation and mEPP amplitude was also reduced. Figure 4C shows significant changes in mEPP amplitude, mEPP half-width, EPP half-width and quantal content (number of quanta released per stimulation). EPP amplitude was not different due to the increase in quantal content. A reduction in mEPP amplitude could be caused by a decrease in post-synaptic AChR expression (but see below). At 6 weeks of age, prior to the onset of weakness, there were no differences in any parameter of neurotransmission measured (data not shown).

Alteration of mEPP duration could indicate a change in AChR current kinetics or be due to changes in muscle membrane input resistance. To address the latter, we directly measured miniature end-plate current (mEPC) using a two-electrode voltage clamp. Supplementary Figure 4d shows example traces from control and M-LAP1^−/−^  *diaphragm* recordings. The mEPCs observed in M-LAP1^−/−^  *diaphragm* were significantly prolonged, with decay time constant (tau) significantly longer compared with littermate controls (Fig. 4D). Of note, not all recordings from M-LAP1^−/−^ mice had prolonged mEPCs. There appeared to be two populations, some of which appeared normal (tau ∼1.3 ms) whilst many had a tau of around 3 ms. We postulated that this is due to expression of the foetal AChR, and for comparison we made recordings from an AChR deficiency mouse model in which neuromuscular AChR current is exclusively via foetal AChR (Cossins *et al.*, 2004). In these AChR deficiency mice, mEPC decay tau had a mean of 2.86 ms which is very similar to the population of M-LAP1^−/−^ recordings with prolonged mEPC decay seen here (Fig. 4D).

### Synaptic levels of AChR are unchanged, but foetal AChRs are up-regulated in M-LAP1^−^^/^^−^ mice

**Figure 5:**
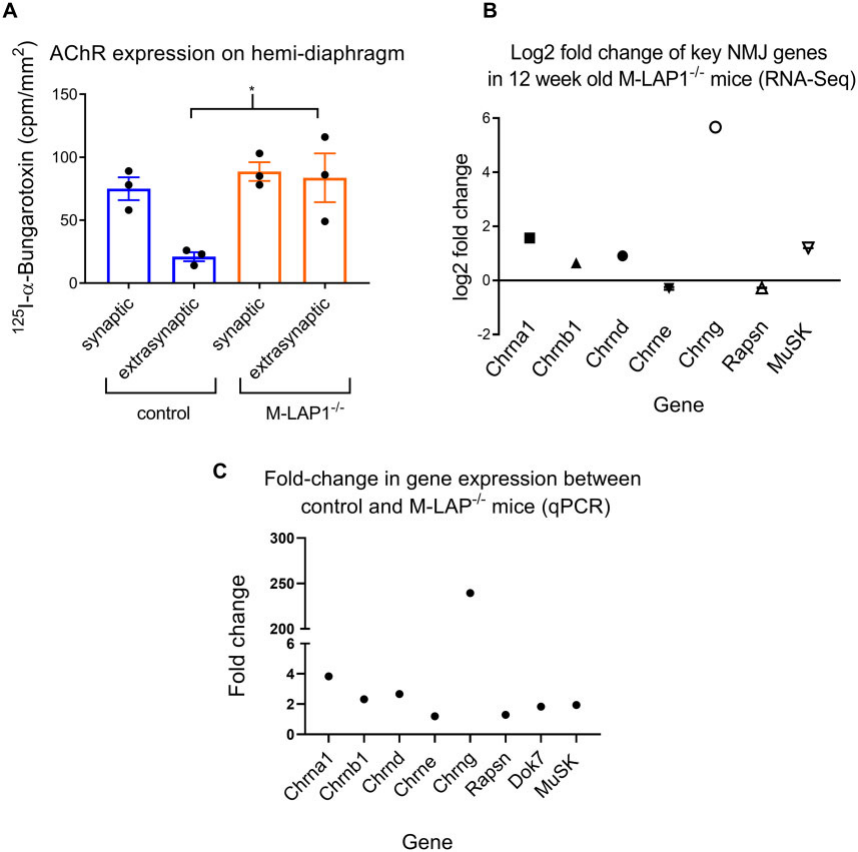
**Analysis of synaptic and extrasynaptic AChR expression, and expression of key NMJ genes in M-LAP1^−/−^ mice and littermate controls.** (**A**) Synaptic and extrasynaptic AChR expression levels in *hemidiaphragm* preparations were measured using ^125^I-α-BuTx. Synaptic levels of AChR are unchanged in the M-LAP1^−/−^ mice, but AChR expression is increased in extrasynaptic regions. Three mice age 12 weeks per group; *P < 0.05. (**B**) RNA-seq analysis of differential expression of key NMJ genes. RNA extracted from EDL muscles from 12 week old M-LAP1^−/−^ mice and littermate controls were compared, three mice per group. (**C**) qPCR analysis of expression of genes encoding the AChR subunits, MuSK, Dok7 and rapsyn. Expression of each gene was compared with expression of *Hprt1*, which was used as a housekeeping gene control. The fold change between the control and M-LAP1^−/−^ mice was then calculated.

The reduction in mEPP amplitude in the mature M-LAP1^−/−^ mice could be due to a reduction in post-synaptic AChR density. To address this, we quantified synaptic and extra-synaptic AChR expression on *hemidiaphragm ex vivo* preparations from 12-week-old M-LAP1^−/−^ mice and littermate controls using ^125^I-α-BuTx. There was no change in synaptic AChR levels in the M-LAP1^−/−^ mice, suggesting that AChR deficiency was not the cause of muscle weakness or of the decreased mEPP amplitude. However, there was an increase in extra-synaptic AChR expression, suggestive of muscle regeneration and/or denervation (Fig. 5A).

From the electrophysiological data above, it seems likely that in at least a proportion of NMJs in weak M-LAP1^−/−^ mice utilize foetal AChR to enable neurotransmission. Foetal and adult AChRs differ from one another by expression of either a γ or an ε subunit, respectively. To confirm up-regulation of the AChR γ subunit in 12 week old M-LAP1^−/−^ mice, and to investigate changes in the expression of the other AChR subunits as well as certain other key genes *Rapsn*, *Dok7* and *Musk*, that are important for NMJ formation and function, we performed RNA-seq using RNA extracted from EDL muscles from three control and three M-LAP1^−/−^ mice. We also analysed the expression of these genes in 6-week old mice, prior to the onset of muscle weakness.

At 6 weeks of age, as expected, the expression of the AChR γ-subunit mRNA was essentially not detectable for both control and M-LAP1^−/−^ mice, being below the cut-off of 1 count per million. At this age, none of the genes studied were differentially regulated [see Supplementary Table 3 for the reads per kilobase per million (RPKM) data for each gene]. By contrast, at 12 weeks of age, the expression of the AChR γ-subunit (*Chrng)* mRNA in M-LAP1^−/−^ mice compared with littermate controls is markedly increased by log2 (5.667) which is ∼50-fold (Fig. 5B). Alignments of the RNA-seq data with the *Chrng* locus obtained from the 12-week-old mice are shown in the UCSC genome Browser in Supplementary Fig. 5. In addition, the AChR-α subunit (*Chrna1*) is upregulated by almost 3-fold (log2(1.567)), Musk is modestly increased by approximately 2.3-fold, and the AChR β- and δ-subunits are increased by around 1.5- to 2-fold. The genes encoding the AChR ε-subunit and rapsyn are both reduced by only 0.8-fold compared with littermate controls, but such a small change is unlikely to be of biological significance (Fig. 5b). Dok7 was not significantly differentially regulated. Utrophin expression was unchanged and dystrophin levels were reduced by 30% (rpkm data shown in Supplementary Table 3).

The changes in gene expression in 12-week old mice identified by RNA-seq were confirmed using qPCR. Gene probes that were used are listed in Supplementary Table 1. The target gene expression for each group was normalized to the expression of internal gene *Hprt1* (Supplementary Fig. 6). These data were then used to calculate the fold change in gene expression in M-Lap^−/−^ mice compared with controls (Fig. 5c). As expected, expression of the foetal AChR γ subunit is almost undetectable in control mice, whereas in M-LAP1^−/−^ mice it is expressed at similar levels to that of other AChR subunit genes, indicating transcription has been switched on. The data from qPCR analysis confirms the results from the RNA-seq data (compare Fig. 5C with Fig. 5B).

### M-LAP1^−/−^ NMJs are fragmented and enlarged, with increased numbers of nuclei

Although the expression of foetal AChRs in weak M-LAP1^−/−^ mice can explain the prolonged mEPPs, EPPs and mEPCs observed above, it is unlikely that γ-subunit expression directly causes the weakness and decrement observed. To gain further insight into how the loss of LAP1 might affect synaptic function, we analysed the morphology of the NMJs in the M-LAP1^−/−^ mice. NMJs were visualized on teased muscle fibres from *soleus*, *diaphragm* and EDL muscles from a 12-week old model and littermate control using 594-α-BuTx. In all muscles tested, the NMJs in the M-LAP1^−/−^ mice were fragmented in comparison to littermate controls (Supplementary Fig. 7). NMJs in EDL muscles showed the most severe fragmentation. The MCK promoter which drives Cre expression is expressed at higher levels in primarily fast-twitch glycolytic muscles such EDL than in slow-twitch oxidative muscles such as soleus (Yamashita and Yoshioka, 1991). Therefore, it may be that LAP1 is more efficiently knocked out in EDL, giving rise to the severe NMJ fragmentation. This muscle was selected for further analyses.

**Figure 6:**
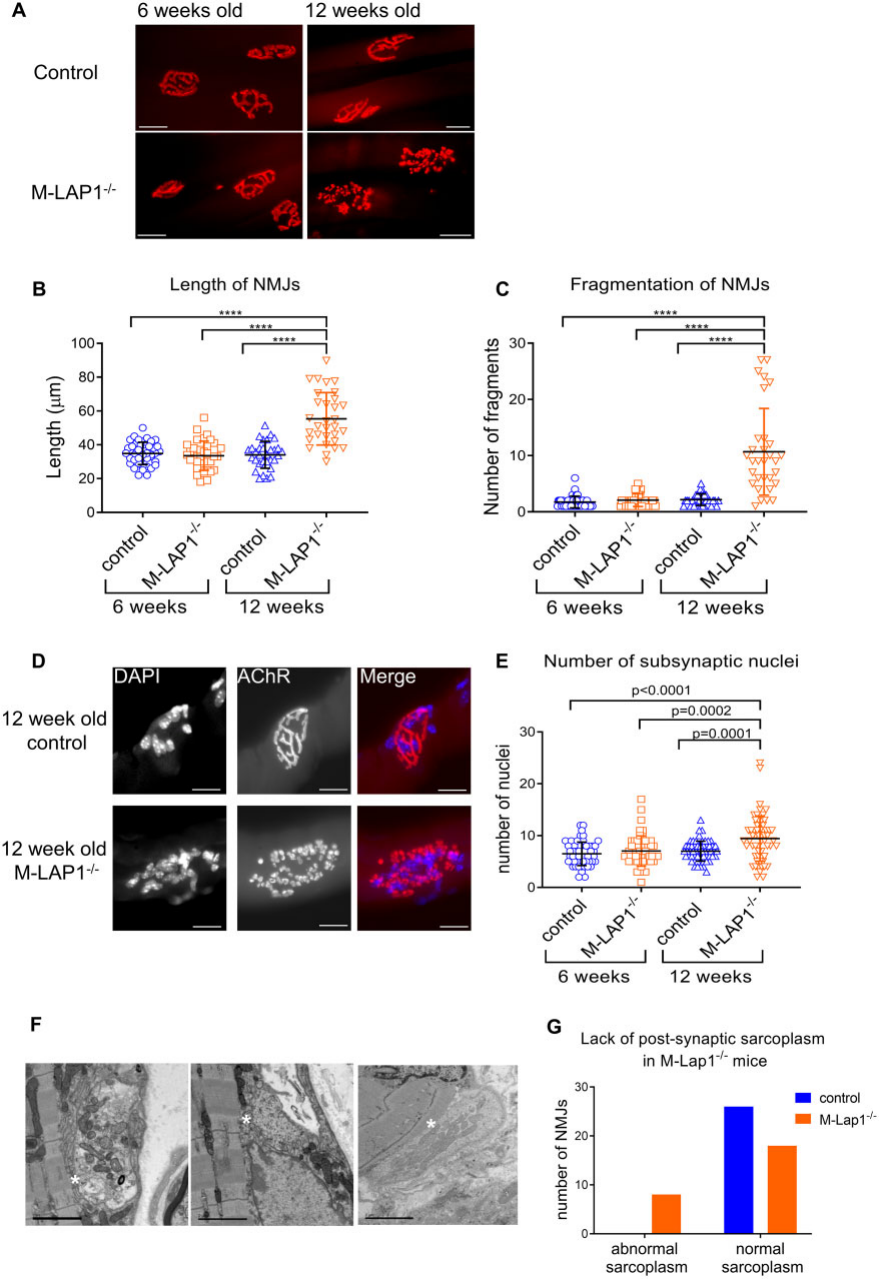
Morphology of NMJs in EDL muscles from M-LAP1^−/−^ mice and littermate controls at 6 and 12 weeks of age. (**A**–**C**) Morphological analysis of NMJs at 6 and 12 weeks of age in EDL muscles from M-LAP1^−/−^ mice and littermate controls. (**A**) The NMJs were visualised by staining AChRs with 594-α-BuTx (red), scale bar=20µm. (**B, C**) Length of NMJs and the number of fragments which made up each NMJ were measured. Each point represents a NMJ. Two mice per group, number of NMJs per mouse: 6 week control 20,20; 6 week M-LAP1^−/−^ 21,16; 12 week control 20,11; 12 week M-LAP1^−/−^ 14,17. ANOVA analysis with Tukey’s multiple comparison, ****P < 0.0001. (**D, E**) Synaptic nuclei number in M-LAP1^−/−^ and control mice EDL muscle. **D** Examples of NMJs from 12 week old control and M-LAP1^−/−^ mice showing the nuclei (left panels, stained with DAPI) and AChRs (middle panels, stained with 594-α-BuTx). Right panels show merged images. In this example, the control NMJ has eight subsynaptic nuclei and the M-LAP1^−/−^ NMJ has 23. Scale bar=20 µm. (**E**) Quantification of number of subsynaptic nuclei in 6 week old and 12 week old control and M-LAP1^−/−^ mice. Each point represents a NMJ. Three mice per group, number of NMJs per mouse: 6 week control 20,18,17; 6 week M-LAP1^−/−^ 19,19,16; 12 week control 23,18,19; 12 week M-LAP1^−/−^ 20,17,20. ANOVA analysis with Tukey’s multiple comparison, *P* values as shown. (**F, G**) Transmission electron microscopy analysis of NMJs in EDL muscle from 12 week-old M-LAP1^−/−^ mice and littermate controls. (**F**) Example electron micrographs showing abnormal endplates in which there is a reduction in the sole plate, meaning that there is very little post-synaptic sarcoplasm and the nerve terminal appears to almost touch the sarcomeres (indicated by white asterisks). Scale bars = 2 µm. (**G**) Contingency graph showing a reduction of sole plate area in some M-LAP1^−/−^ mice but not in littermate controls. Number of NMJs analysed per group = 26, three mice per group. Chi-square (and Fisher’s exact test); *P* = 0.0042.

The size and fragmentation of the NMJs were studied in detail. At 6 weeks of age, before mice showed weakness in our strength test, NMJs on EDL muscle fibres from model mice appeared normal and were indistinguishable from littermate controls in size and fragmentation (Fig. 6A). The average length of NMJs for M-LAP1^−/−^ and control mice was 35 µm (ranging from 18 to 56 µm) and comprised of a mean of two fragments (Fig. 6B, C). At 12 weeks of age, the morphology of NMJs in control mice was unchanged. In marked contrast, most of the NMJs in 12-week-old M-LAP1^−/−^ mice had abnormal morphologies. The average length increased to ∼55 µm, with a much larger variability from 30 µm to 90 µm. The NMJs in weak M-LAP1^−/−^ mice were very fragmented (average of ∼11 fragments per NMJ compared with two for the controls), and comprised of small individual boutons or islands, rather than long interconnected channels (Fig. 6A–C). Even so, the axon termini were generally still in good registration with post-synaptic AChR staining as seen in Supplementary Fig. 8a. For both control and M-LAP1^−/−^ mice, the occasional NMJ showed evidence of presynaptic sprouting (Supplementary Fig. 8b, sprouting indicated by red arrows).

We next analysed the number of synaptic nuclei in M-LAP1^−/−^ mice. Synaptic myonuclei which reside directly underneath the synapse are transcriptionally specialized and express NMJ proteins such as the AChR ε subunit (Sanes and Lichtman, 2001; Schaeffer *et al.*, 2001). These nuclei are tethered in place by transmembrane actin-associated nuclear (TAN) lines, a complex of inner and outer nuclear membrane proteins (including Nesprins) which interact with actin in the cytoplasm (Grady *et al.*, 2005). LAP1 and TorsinA control the assembly of TAN lines (Saunders *et al.*, 2017), therefore we reasoned that loss of LAP1 in the M-LAP1^−/−^ mice might lead to a gradual loss of subsynaptic nuclei, leading to a reduction in synaptic gene expression and thus providing a mechanism by which the mice become weak. We counted the number of nuclei at each NMJ in single muscle fibres. At 6 weeks of age, there was no significant difference in the number of nuclei between M-LAP1^−/−^ and littermate controls (mean 7 and 6.5, respectively, Fig. 6D, E). However, at 12 weeks of age, contrary to expectations, many of the NMJs in the M-LAP1^−/−^ mice had an increase in subsynaptic nuclei number (mean M-LAP1^−/−^ = 9.5; mean control = 7.0), with a much larger variation in number with some NMJs having more than 20 nuclei (Fig. 6D, E).

### Ultrastructural abnormalities in LAP1 KO NMJs

Using transmission EM, we explored the ultrastructure of 12-week-old M-LAP1^−/−^ mice and littermate controls (two mice per group). There were no significant changes in the width of the synaptic cleft, pre- and post-synaptic membrane length or area, synaptic index (ratio of post-synaptic membrane length: pre-synaptic membrane length), number and length of primary folds, number of secondary folds, or number of vesicles/pre-synaptic area (see Supplementary Table 4). However, some of the M-LAP1^−/−^ NMJs were abnormal. In 30% of NMJs, there was a qualitative reduction in the sole plate (this is the sarcoplasm between the post-synaptic membrane and sarcomeres) indicated by asterisk in Fig. 6F. In some of these instances, there was a total absence of primary folds. This was never observed in control mice. The data are shown in the contingency graph (Fig. 6G).

### Evaluation of neuromuscular transmission in other nuclear envelopathies

Finally, to investigate whether neuromuscular transmission abnormalities could be a more general feature of nuclear envelopathies, we conducted neurophysiological studies in three individuals (cases 3–5) with muscular dystrophy. One had LGMD due to *LMNA* mutations and two had Emery–Dreifuss Muscular Dystrophy due to *EMD* and *LMNA* mutations, respectively. The results showed no significant decrement to repetitive nerve stimulation from proximal and distal muscles in any of the three cases. Single fibre examination showed isolated pairs with raised jitter but overall no blocking was recorded. Details are shown in Table 2.

**Table 2:** Electromyography data from Limb girdle muscular dystrophy cases

	Individual 3	Individual 4	Individual 5
Age (years)	22	45	29
Diagnosis	Limb girdle muscular dystrophy	X-linked Emery–Dreifuss Muscular Dystrophy	Autosomal Dominant Emery–Dreifuss Muscular Dystrophy
Mutation	*LMNA* c.1357C>T, p. Arg453Trp	*EMD* c.266-3A>G Splice site	*LMNA* c.1072G>A p.Glu358Lys
Decrement on repetitive nerve stimulation (muscles tested)	No (*extensor digitorum brevis*, *abductor digiti minimi*, *trapezius, nasalis*)	No (*abductor digiti minimi*, *trapezius*)	No (*abductor digiti minimi*, *trapezius*)
Single fibre EMG (muscles tested)	Subtly abnormal, no blocking (*extensor digitorum communis*)	Jitter values ranged from normal to high, no blocking (*extensor digitorum communis*, *abductor digiti minimi*)*	No blocking or jitter (*extensor digitorum communis*)
Routine EMG	Typical myopathic features	Typical myopathic features and high amplitude increased duration motor unit potentials	ND

ND = not done.

## Discussion

Here, we describe a congenital myasthenic syndrome due to a mutation in *TOR1AIP1*, which encodes LAP1, a nuclear envelope protein. We used an M-LAP1^−/−^ mouse model in which both isoforms of the gene are conditionally knocked out in striated muscle, to gain insight into the pathogenic molecular mechanisms. In accordance with neurophysiology recorded in two affected siblings with the *TOR1AIP1* mutation, RNS of M-LAP1^−/−^ mouse muscle resulted in a decrement of CMAP indicative of impaired signal transmission at the NMJ. The NMJs in the mice were enlarged and fragmented, but post-synaptic AChR expression levels in muscles tested were unchanged. In addition, in electrophysiological measurements of endplate function mEPPs and EPPs were prolonged, which could be explained by an observed up-regulation of the AChR γ subunit. Thus, we identify a new class of protein in which defective function leads to impaired synaptic transmission.

Rare mutations in *TOR1AIP1* have been described giving rise to a limb girdle muscular dystrophy phenotype with cardiac involvement, but with no report of defective neuromuscular transmission. In our cases, the train of the decremental response on RNS gives an envelope or U shape and this is typical for myasthenia (see the illustrative trace from the patient, Fig. 1C). Other wasting diseases such as motor neuron disease more often produce a pattern of continuous reduction of the amplitude of the CMAP to repetitive motor nerve stimulation. Standard needle electromyography performed on the *deltoid* muscle in individual 1 showed a full interference pattern of motor unit action potentials without fibrillations or fasciculations, thus excluding denervation. In individual 2, the standard needle electromyographic sampled the *extensor digitorum communis* and this was normal, so there is no pathology other than CMS to account for the decrement.

In four previous reports of *TOR1AIP1* mutations in a total of 12 individuals, clinical features range from a relatively mild muscular dystrophy to a severe multi-system phenotype (18–20, 30). This phenotypic spectrum seems to be at least in part determined by the location of the mutations within the gene. For example, two siblings with a homozygous frameshift, c.186delG p. Glu62fs*, that only affects LAP1B presented with a relatively mild clinical phenotype including limb-girdle muscular dystrophy with atrophy, contractures and a dilated cardiomyopathy (Kayman-Kurekci *et al.*, 2014). At the most severe end of the spectrum a progressive multi-system disease with early lethality is caused by mutation c.961C>T, p. Arg321* which results in loss of both LAP1B and LAP1C isoforms (Fichtman *et al.*, 2019). By contrast, the two cases we describe here with a homozygous c.127delC, p. P43fs* mutation (which is predicted to only affect LAP1B), did not have any cardiac involvement. Thus, the evidence to date suggests mutations specific to LAP1B, the muscle isoform, give a less severe phenotype.

A clinical feature in common for *TOR1AIP1* mutations is a muscular dystrophy, which is also present in the conditional M-LAP1^−/−^ mouse model (Shin *et al.*, 2013). Since the cases we describe presented with myasthenic features, we used this mouse model to investigate the post-synaptic role of LAP1 in the structure and function of the NMJ. It should be noted that both isoforms of the protein are conditionally knocked out in the mouse model used here, whereas in our patients, only LAP1B expression is affected. However, we reasoned that, because LAP1B is the only detectable isoform present in myonuclei [data shown here and by Kayman-Kurekci *et al.* (2014)], this model reflects the human situation. First, EMG showed that the mice, when weak, had decrement on RNS. Examination of neuromuscular signalling in greater detail showed a reduction in mEPP amplitude and prolonged mEPP, EPP and mEPC. However, EPP amplitude was not reduced, which is potentially inconsistent with the observed fatigable weakness. EPP amplitude was maintained at steady-state stimulation (1 Hz) as a consequence of increased quantal content. This phenomenon of homeostatic compensation by increased quantal content (Plomp *et al.*, 1992) has been observed in human and rodent model myasthenia gravis induced weakness (Plomp *et al.*, 1995; Viegas *et al.*, 2012) and other transgenic models of muscle weakness (Rozas *et al.*, 2011; van der Pijl *et al.*, 2016). At faster stimulation, this homeostatic mechanism is likely to diminish (anecdotally observed as EPP amplitude rundown at 50 Hz in our mouse model). The loss of higher quantal content enhancement at high stimulation frequency is likely due to the depletion of specific vesicle pools (Wang *et al.*, 2016) and is seen in other myasthenia models (Rozas *et al.*, 2011). At higher physiological burst frequencies, EPP amplitude is not likely to be maintained leading to a sub-threshold post-synaptic depolarization with subsequent failure in muscle action potential generation. This mechanism underlying myasthenic weakness has previously been proposed for autoimmune myasthenia gravis (Wang and Rich, 2018). Reduced mEPP amplitude can also be indicative of a reduced number of AChRs on the post-synaptic membrane, but this is unlikely to be the case here, since measurement of synaptic AChR was found to be the same in both M-LAP1^−/−^ mice and controls. However, it is possible that the density of AChRs in the M-LAP1^−/−^ mice might be reduced, particularly since the NMJs are enlarged and fragmented. Although fluorescent labelling of AChRs suggests normal overall density, this might not be true at an ultrastructural level where there might be localized reductions in AChR expression. In addition, the localization of AChRs underneath the active zones might be altered. Detailed immunogold labelling and electron microscopic analysis would be required for this type of analysis.

An alternative explanation for the electrophysiological findings is an up-regulation of foetal AChRs, which have a lower single-channel conductance than the adult AChR (39pS and 59pS respectively) and longer open times (10.4 and 5.3 ms, respectively) (Mishina *et al.*, 1986). We demonstrate a substantial re-expression of γ subunit mRNA using qPCR, thus confirming the likely presence of foetal AChR in the weak 12-week-old M-LAP1^−/−^ mice. We also observed a small increase in expression of the AChR α-, β- and δ-subunits as well as Musk. The results suggest some loss occurring in the control of the synapse-specific expression.

Up-regulation of the AChR γ subunit and extra-synaptic expression of AChRs (which we also observe) are known to be observed when muscle is denervated (Miledi, 1960; McMahan *et al.*, 1980). However, we saw very little evidence for denervation on whole muscle fibres co-stained for AChRs, synaptophysin and neurofilament at 12 weeks of age when the mice were weak. Pre- and post-synaptic staining was in good apposition at almost all NMJs. The fragmentation of NMJs which we observed (discussed below) is also atypical of denervation. It seems likely that increased AChR γ here is caused by ongoing myoblast differentiation as muscle is regenerating, or there could be a loss in the control of the synapse-specific expression by the subsynaptic nuclei, but it is unclear whether LAP1 directly impacts on the expression of the AChR subunits during this process.

To further investigate why there is defective neuromuscular signalling in the mouse model, we looked at the morphology of the NMJs. The main morphological change in the weak M-LAP1^−/−^ mice was the fragmentation of the NMJs into many small boutons or islands. This is characteristic of NMJs in mouse models of muscle regeneration (Couteaux *et al.*, 1988; Li and Thompson, 2011) (Hansen-Smith, 1986) and has also been observed in the mdx mouse model and a canine model of Duchenne muscular dystrophy where muscle fibres degenerate and regenerate (Lyons and Slater, 1991; Banks *et al.*, 2009; Pratt *et al.*, 2015; Haddix *et al.*, 2018), and in aged mice (Li *et al.*, 2011). It was accompanied by an increase in synaptic nuclei, which has also been observed in mdx mice (Pratt *et al.*, 2015).

Given the similarity in NMJ structure between the mdx mouse and the M-LAP^−/−^ mice, it was possible that the depletion of LAP1 might affect the expression of dystrophin and/or utrophin. However, RNAseq showed no change in utrophin levels and a decrease of 30% in dystrophin expression, which is unlikely to contribute to the pathology since mdx mice expressing a transgene at 70% WT levels appear essentially normal (Phelps *et al.*, 1995). That notwithstanding, the M-LAP^−/−^ mice do show quite a severe muscular dystrophy by week 12. It is therefore possible that the NMJ morphological changes could be secondary to muscle degeneration/regeneration, and not a direct result of the absence of LAP1. This could be ascertained by analysing individual muscle fibres to see whether fragmented NMJs coincide with strings of central nuclei, which are indicative of muscle regeneration. It would also be of interest to generate a mouse model in which only the muscle-specific LAP1B isoform is knocked out. This would be more representative of the mutation found in our patients, might have a less severe dystrophy and would allow us to tease apart the myasthenic features of the disease from the dystrophic phenotype.

Contradictory results make it unclear whether NMJ fragmentation in itself impairs NMJ function [reviewed in Slater (2020)]. For example, aged mice have fragmented NMJs but neurotransmission is not compromised (Willadt *et al.*, 2016). In addition, one study in mdx mice shows no abnormal neurotransmission (Lyons and Slater, 1991), whereas others have reported a reduction in MEPP amplitude and increased quantal content (Nagel *et al.*, 1990; Carlson and Roshek, 2001; van der Pijl *et al.*, 2016). Considering these data, we cannot be sure that the altered neurotransmission observed here is a direct result of NMJ fragmentation.

To further explore possible pathogenic mechanisms, we used electron microscopy to analyse the morphology of NMJs at a higher resolution. On average, there was no significant difference in ultrastructure between M-LAP1^−/−^ mice and controls in the parameters measured. However, around 30% of NMJs in the M-LAP1^−/−^ mice showed a reduction in the sole plate (the sarcoplasm between the post-synaptic membrane and sarcomeres), and some of these NMJs also had a reduced number of secondary folds. These structural defects could contribute to impaired signalling in a similar fashion to defects in the AGRN-LRP4-MUSK-DOK7 pathway.

Defective neurotransmission has not been reported in nuclear envelope-associated muscular dystrophies, and so we assessed neurophysiology (EMG) on one patient with LGMD (*LMNA* mutations) and two with Emery–Dreifuss muscular dystrophy, the archetypal nuclear envelopathy (one patient had *LMNA* mutations and the other had *EMD* mutations). No decrement on RNS was observed in these patients. A mouse model of autosomal dominant Emery–Dreifuss muscular dystrophy in which the gene encoding LaminA/C is ablated, has a more severe phenotype than the M-LAP1^−/−^ mouse. The NMJs in that model do show some structural change, but to a lesser extent that we describe here in the M-LAP1^−/−^ mouse. In addition, there is a loss of synaptic myonuclei in the autosomal dominant Emery–Dreifuss muscular dystrophy mouse model, which is in direct contrast with the increase we observe in the M-LAP1^−/−^ mouse (Méjat *et al.*, 2009). Thus, even though LAP1 and LaminA/C interact, the pathogenic mechanism appears different.

In summary we describe here an autosomal recessive congenital myasthenia caused by mutations in *TOR1AIP1*, encoding a nuclear envelope protein. Our results suggest LAP1B has a specific role at the NMJ, since mutations in other nuclear envelope proteins with which LAP1 is thought to interact such as Emerin or LaminA/C did not show impaired neuromuscular transmission as measured by EMG. It is of note that the cases we present have no cardiac involvement. Our data and the relatively late onset of the disorder would suggest a disorder associated with destabilization of synaptic structure rather than a direct effect of components of synaptic transmission. Characteristics of the disorder we describe here, such as the marked increase in AChR γ-subunit mRNA expression (in the absence of denervation) and the loss of synaptic structure, may have implications for other pathologies where the NMJ is involved. In particular, the results indicate that patients with mutations within *TOR1AIP1* should benefit from therapy with cholinesterase inhibitors.

## Supplementary material

Supplementary material is available at *Brain Communications* online.

## Supplementary Material

fcaa174_Supplementary_Data

## Abbreviations

AChR =: acetylcholine receptor
α-BuTx =: α-Bungarotoxin
CK =: creatine kinase
CMAP =: compound muscle action potential
EDL =: *Extensor digitorum longus*
LAP1 =: lamin-associated protein 1
LRP4 =: low-density lipoprotein receptor-related protein 4
mEPC =: miniature end-plate current
m(EPP) =: (miniature) end-plate potential
MuSK =: muscle-specific tyrosine kinase
NMJ =: neuromuscular junction
QMG =: quantitative myasthenia gravis
