# Supplementary material for: Congenital myasthenic syndrome due to a TOR1AIP1 mutation: a new disease pathway for impaired synaptic transmission
Source: Brain Commun. 2020 Oct 18;2(2):fcaa174. doi: 10.1093/braincomms/fcaa174 (PMC7660151; doi:10.1093/braincomms/fcaa174)
Supplement: fcaa174_Supplementary_Data [file fcaa174_supplementary_data.docx]

**Supplementary Methods**

**Electromyography in M-LAP1^-/-^ mice**

Anaesthesia was induced using intraperitoneal Hypnorm® (Fentanyl 0.1 mg/kg and Fluanisone 3 mg/kg) (Vetpharma Ltd.) and maintained using inhaled Isoflurane (1.25-1.75%) / Oxygen. Mouse rectal temperature was maintained between 37 °C and 38 °C. The sciatic nerve was stimulated at the level of the hip. Compound Muscle Action Potentials (CMAPs) were recorded from *gastrocnemius* muscles (Dual bio amp/stimulator and Powerlab 4/25, AD Instruments). A train of ten stimuli supramaximal was applied at frequency ranging from 1 to 20 Hz. Decrement was analysed at varying stimulation frequencies, results of three test series were averaged (pClamp 9, Molecular Devices).

**Electrophysiology using M-LAP1^-/-^ mouse hemi-*diaphragm* preparations**

Following euthanasia, phrenic nerve/hemi-*diaphragm* preparations were dissected and bathed in Krebs solution, containing 2.5 mM CaCl2 and bubbled with 95% O2 / 5% CO2 (Cossins *et al.*, 2004). μ-Conotoxin GIIIB (2.5 μM, Peptide Institute Inc.) was added the bath for 30 minutes to block muscle contractions. The excess unbound toxin was washed out before recordings started. Recordings were made at 22-23 °C.

The phrenic nerve was pulled into a suction electrode, which was coupled to a pulse generator, with an associated stimulus isolation unit (GRASS instruments S48 square pulse stimulator). Recording electrodes were connected to an Axoclamp 900A amplifier (Molecular Devices). Data signals passed through a Humbug 50 Hz noise eliminator (Quest Scientific via Digitimer). Signals were continuously digitised at 10 kHz sampling rate and filtered at 4 kHz, using Axon Digidata 1322A interface, controlled by pClamp 10 software (Molecular Devices). Depolarisations at the endplate were recorded intracellularly using a single borosilicate glass micropipette electrode. Electrodes were pulled by a programmable P-97 microelectrode puller (Sutter Instruments, Novato, CA) and filled with 3M KCl (10–30 MΩ). The recording electrode was positioned above endplate regions, as visualised by stereomicroscope (Olympus BX51WI) under micromanipulator control (Scientifica).

Impalement adjacent to an endplate was indicated by fast rise time of miniature endplate potentials (mEPPs), defined as less than 2 ms. To evoke an endplate potential (EPP), the phrenic nerve was stimulated via a suction electrode.

Each mEPP and EPP was detected via template or threshold searching in Clampfit 10 software. Analysis was performed blinded to treatment group. All mEPP and EPP amplitude measurements were adjusted for deviation of a resting membrane potential of –80 mV (Katz and Thesleff, 1957). Mean mEPP amplitude was derived from averaging 20-40 mEPPs per endplate. Mean EPP amplitude per endplate was derived from averaging a train of 20 EPPs evoked at 1 Hz.

Mean quantal content (m) was calculated per endplate, by dividing the mean amplitude of EPP (stimulated at 1 Hz) by the mean amplitude of mEPP, using the formula:

m = mean (AMPEPP(corrected)) / mean (AMPmEPP)

Where AMPEPP(corrected) is the corrected amplitude of the EPP and AMPmEPP is the amplitude of the mEPP. In the above calculation, EPPs (evoked at 1 Hz) were corrected for non-linear summation, using the following formula:

AMP_(EPP (corrected)) = (AMP_(EPP(measured)))/(((1 - 0.8 AMP_(EPP(measured) )))⁄( E))

In the above formula, E is the driving force and was assumed to be 80 mV, and 0.8 was arbitrarily used as the correction factor for mouse endplates (Linder and Quastel, 1978; McLachlan and Martin, 1981).

For two-electrode voltage-clamp recordings of mEPC and EPC, two glass electrodes (3M KCL, ~20 MOhm) were inserted into the same fibre in close proximity in region of muscle endplates visualised using 20x water immersion objective. Two-electrode voltage clamp was achieved via Axoclamp 900A amplifier (pClamp 10 and Axoclamp 900 Commander, Molecular Devices). Clamp gain was maximised to achieved rapid voltage clamp before oscillation and compensation of greater the 95% was achieved, such that only a deflection of < 1 mV remained during stimulation and recording of an EPC during voltage-clamp. Data were filtered at 0.8 kHz and sampled at 10 kHz prior to storage. mEPC Events were detected by template recognition (pClamp 10), at least 20 events were averaged for each fibre recorded.

**Immunofluorescence staining of endplate regions from mouse muscle**

*Extensor digitorum longus* (EDL), *soleus* (SOL) and *diaphragm* muscles were dissected in Kreb’s buffer and pinned on Sylgard. Muscles were fixed for 30mins at RT in Kreb’s buffer containing 1% paraformaldehyde (TAAB Laboratory Equipment). AChRs were visualised by incubating muscle with 594-α-BuTx diluted 1:100 for 1 hour at room temperature. For neurofilament and synaptophysin staining, bundles of muscle were teased apart and dehydrated by incubation in ice cold ethanol (2 minutes) and ice-cold methanol (5 minutes). Tissue was permealised in PBS containing 0.1% triton for 5 minutes at RT. Muscle was washed for 2 hours in PBS and incubated overnight at 4ºC in PBS containing 3%BSA, chicken anti-neurofilament heavy chain antibody (Abcam plc, 1:1000) and rabbit anti-synaptophysin Ab-4 (Fisher Scientific, 1:100). Samples were washed extensively with PBS and incubated for 3 hours at RT with Alexa Fluor® 488-conjugated secondary antibodies (Invitrogen, 1:500). Samples were washed overnight, mounted using Confocal Matrix (Micro Tech Lab) and then coded so that the image acquisition and analysis were carried out blinded. Stained muscles were visualised using an Olympus IX71 wide field fluorescence microscope. Images were captured using Simple PCI (Digital Pixel) and analysed using ImageJ. Number of mice used for each analysis was as described in the figure legends.

**Quantification of endplate AChR**

One hemi-*diaphragm* from each mouse was trimmed and pinned out on Sylgard 184 (Dow Corning), and incubated for 2 h at room temperature in Kreb's buffer (118 mM NaCl, 4.7 mM KCl, 1.2 mM MgSO_4_·7H_2_O, 1.2 mM KH_2_PO_4_, 24.9 mM NaHCO_3_, 11.1 mM D-glucose and 2.5 mM CaCl_2_) containing ^125^I-α-BuTx (Perkin Elmer). The hemi-*diaphragm* was rinsed 3×5 min with Kreb's buffer and then washed overnight at 4°C with Kreb's buffer. The tissue was fixed in 2.5% glutaraldehyde in 80% Kreb's buffer for 2 hours at room temperature and then washed 3×5 min with H_2_O. The *diaphragms* were stained for acetylcholinesterase and the region containing endplates were excised, the dimensions measured, and radioactivity counted in a gamma counter. Two equivalent lengths of muscle devoid of endplates were also measured and counted.

**RNA-seq analysis**

RNA-sequences were aligned to the genome using STAR (Spliced Alignment to a Reference). Reads were counted using featureCounts and analysed using edgeR (Bioconductor). Data was filtered to exclude genes with <1 count per million for at least three samples. RNA composition was adjusted using calcNormFactors, and the coefficient of variation and dispersion were estimated. TopTags was used to obtain log-fold change (logFC) data. Bigwig files were created from BAM files for viewing in the UCSC genome browser.

**qPCR analysis**

**Supplementary Table 1.** List of qPCR gene probes (Life Technologies)

| **Gene name** | **Taqman assay ID** |
| --- | --- |
| *Hprt1* | Mm01324427_m1 |
| *Chrna1* | Mm00431629_m1 |
| *Chrnb1* | Mm00680412_m1 |
| *Chrnd* | Mm00445545_m1 |
| *Chrne* | Mm00437411_m1 |
| *Chrng* | Mm00437419_m1 |
| *Rapsn* | Mm00485539_m1 |
| *Musk* | Mm01346929_m1 |
| *Dok7* | Mm00554697_m1 |

Each biological sample was run in duplicate, using cDNA from three control mice and three M-LAP1^-/-^ mice. cDNA was diluted an appropriate amount. 10μl reactions were set up using TaqMan Gene Expression Master and the relevant gene probe, and were amplified using a QuantStudio7 real time PCR system. The same threshold was set for each sample, and CT values were measured. The mean *Hprt1* CT for each sample was calculated, and was subtracted from each target gene CT value to give ΔCT. The data were transformed using the equation 2^-ΔCT^ and mean±sd values were calculated. One-tailed unpaired Welch’s t test analysis was performed to analyse differences in target gene expression between control and M-LAP1^-/-^ mice.

**Supplementary Figures**

**Supplementary Figure 1**

**
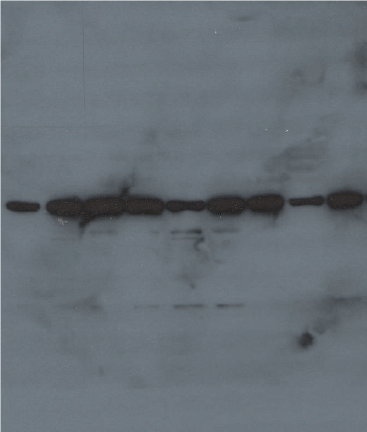

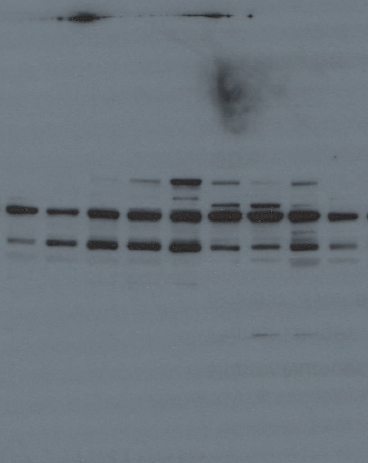
**

62kDa

38kDa

49kDa

92kDa

Molecular weight markers

C

I

I

C

*

**

Antibody

LAP1

α-tubulin

**Supplementary Figure 1 - Full size western blots.** The full size western blots shown in Figure 2c are shown along with the SeeBlue molecular weight markers. I = Individual 1, C = Control. *=LAP1B and ** = LAP1C. The lower band detected with the LAP1 antibody is non-specific.

**Supplementary Figure 2**


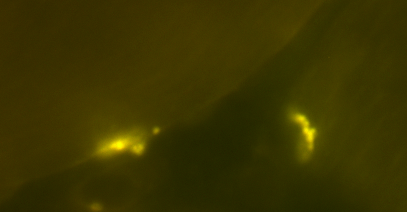

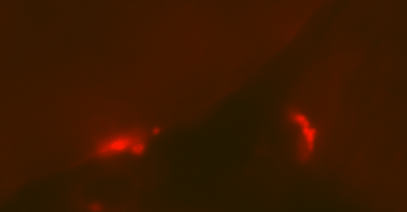

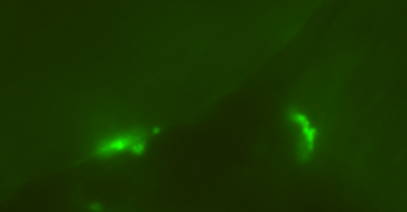


AChR

AChE

Merge

**Supplementary Figure 2 – AChR and acetylcholine esterase expression in muscle biopsy.** AChR was labelled with 594-α-BuTx (red) and acetylcholine esterase (AChE) was labelled with 488-Fasciculin (green). The proteins colocalise.

**Supplementary Figure 3**


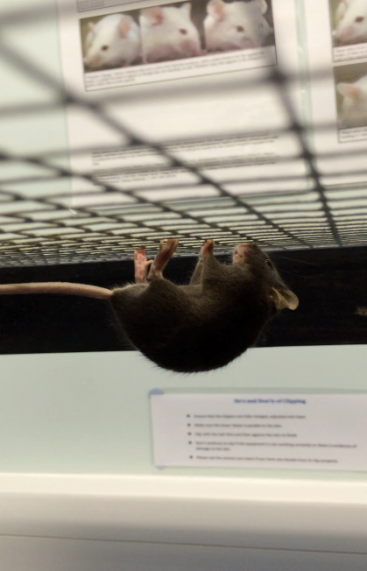


Weight of mice

**B**

**A**

**Supplementary Figure 3 – M-LAP1^-/-^ mouse information.** **A** Photograph showing the hang test used to determine the strength of each mouse. The length of time a mouse could hang upside-down on a wire mesh was measured. The cut-off time was 10 minutes. **B** Growth curves of male and female M-LAP1^-/-^ mice and littermate controls.

**Supplementary Figure 4**

**A**


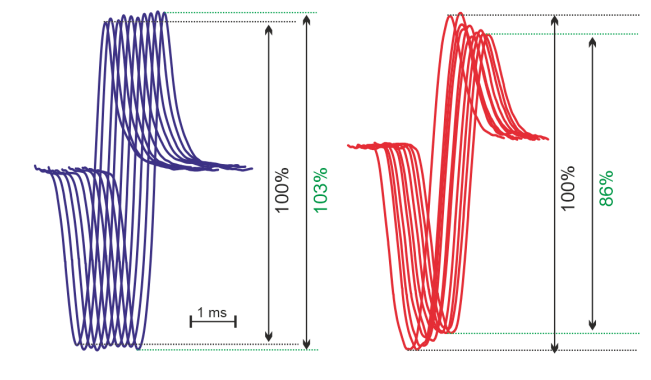


Compound muscle action potential (mV)

Control M-LAP1^-/-^

Time (ms)

**B**

CMAP amplitude (% of 1st stimulation)

6 week old controls 9 week old controls 12 week old controls

6 week old M-LAP1^-/-^ 9 week old M-LAP1^-/-^ 12 week old M-LAP1^-/-^

Stimulation number


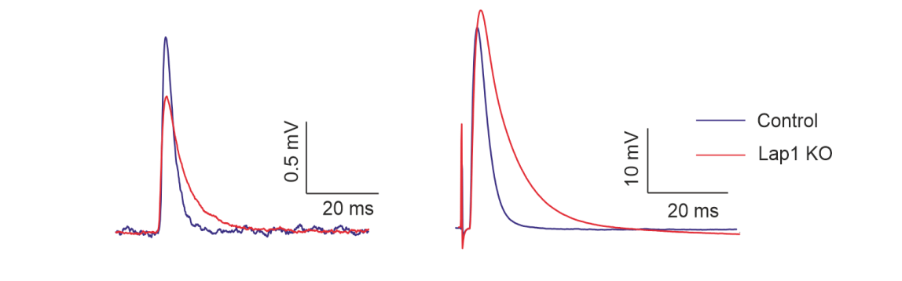


Membrane potential (mV)

mEPP EPP

Time (ms)

Control

M-LAP1^-/-^

Control M-LAP1^-/-^


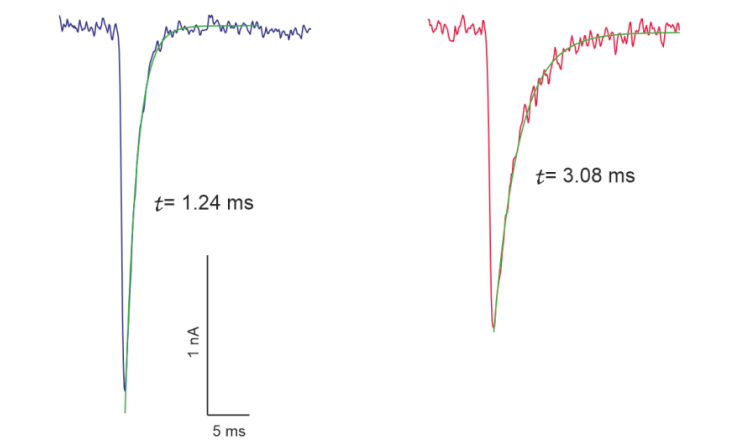


Current amplitude (nA)

**C D**

Control M-LAP1^-/-^


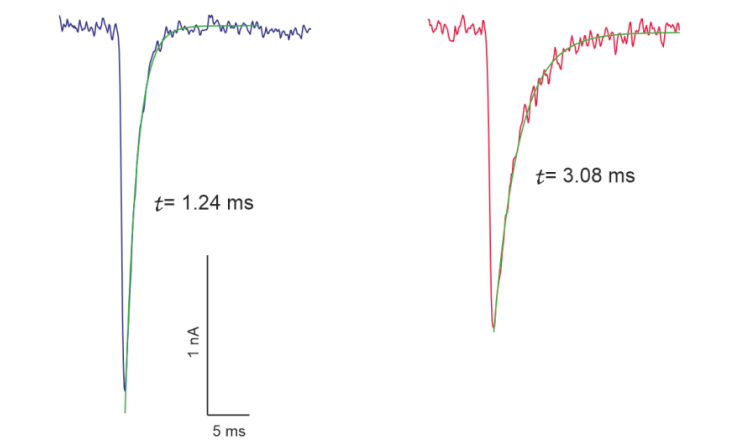


Current amplitude (nA)


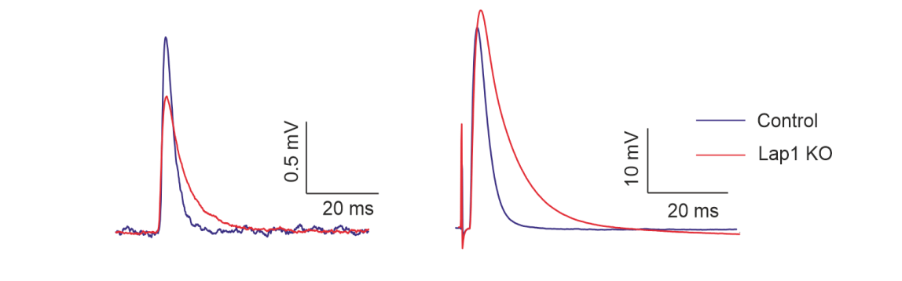


Membrane potential (mV)

mEPP EPP

Time (ms)

Control

M-LAP1^-/-^

**Supplementary Figure 4 – Example electrophysiology traces.**

**A** CMAP decrement on repetitive nerve stimulation in mouse gastrocnemius muscle in a control and a M-LAP1^-/-^ mouse. **B** A different representation of the same EMG data as in Figure 4b showing successive changes in CMAP amplitude over a train of 10 stimuli at various different stimulation frequencies. Each value is the mean, and is calculated as a percent of the initial CMAP value. Mice aged 6, 9 and 12 weeks were analysed (n=3 mice control 6 and 12 weeks and M-LAP1^-/-^ 6 weeks; 4 mice control 9 weeks; 7 mice M-LAP1^-/-^ 9 and 12 weeks). **C** Representative traces of mEPPs and EPPs from a control mouse and a M-LAP1^-/-^ mouse. **D** Endplate current recordings showing reduced amplitude and prolonged signal in M-LAP1^-/-^ mouse compared with control.

**Supplementary Figure 5**


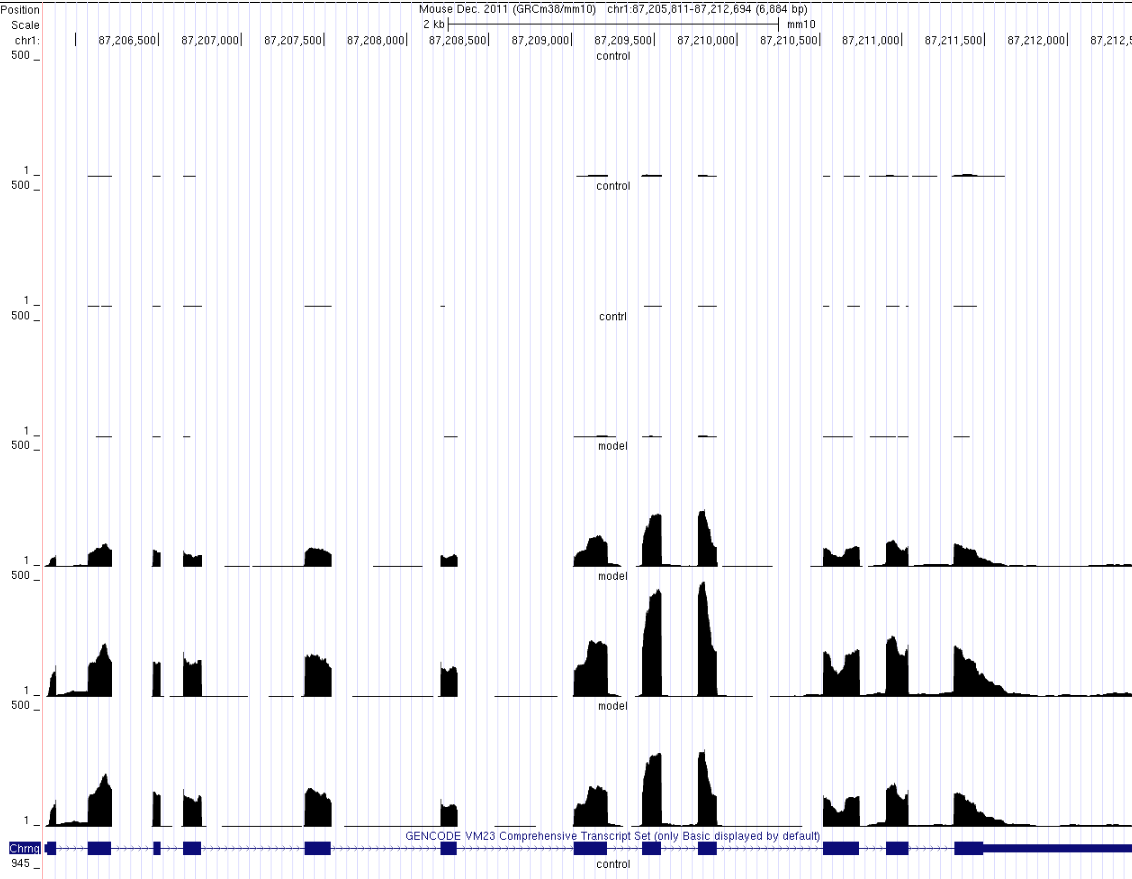


Controls

M-LAP1^-/-^

*Chrng*

**Supplementary Figure 5 – Alignment of RNA-sequence reads at the *Chrng* gene locus**. Alignment of RNA-seq reads from three 12 week old control mice and three 12 week old M-LAP1^-/-^ mice with the *Chrng* gene locus using STAR, and viewed on the UCSC genome browser. The exons of the gene are depicted at the bottom of the figure in blue.

**Supplementary Figure 6**

*

*

Gene

**Supplementary Figure 6 - qPCR analysis of expression of genes encoding the AChR subunits, MuSK, Dok7 and rapsyn.**

mRNA expression of the AChR subunits, MuSK, Dok7 and rapsyn for control and M-LAP1^-/-^mice were measured using qPCR and compared with housekeeping gene *Hprt1*. Three mice per group.

**Supplementary Figure 7**

12 week old control


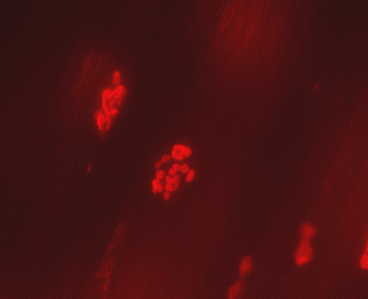


12 week old M-LAP1^-/-^

*Diaphragm*


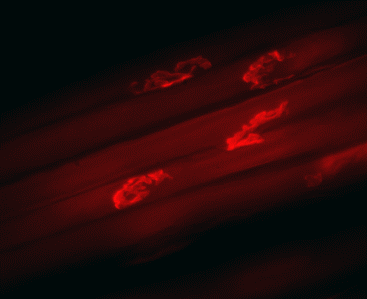

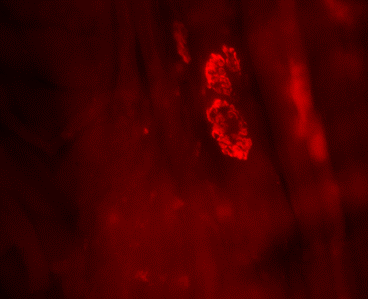


*Soleus*


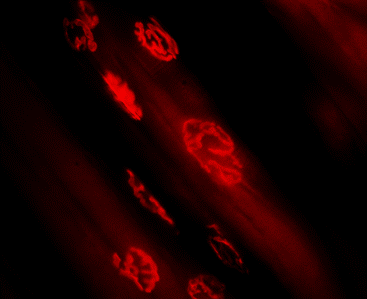

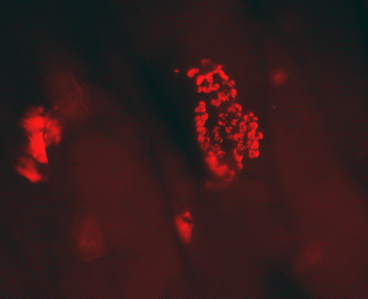


EDL


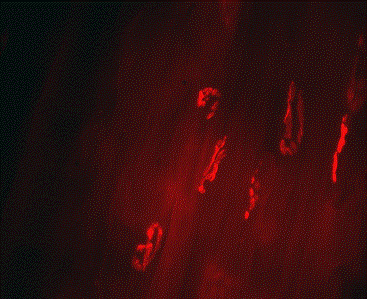


**Supplementary Figure 7 – Representative examples of NMJs of 12 week old M-LAP1^-/-^** **mice** **and littermate controls.** Teased muscle fibres were stained with 594-α-BuTx to visualise AChRs. Scale bar = 20μm

**Supplementary Figure 8**

**A**


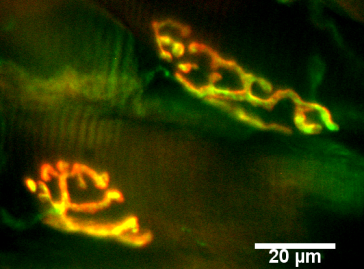

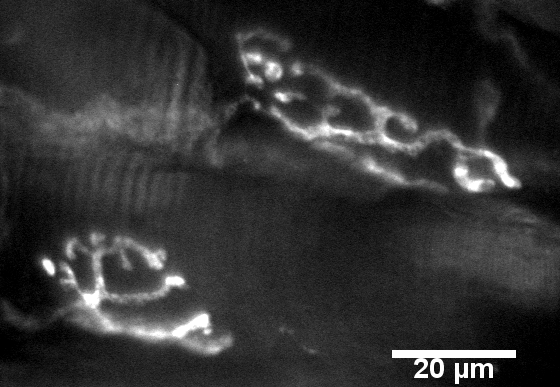

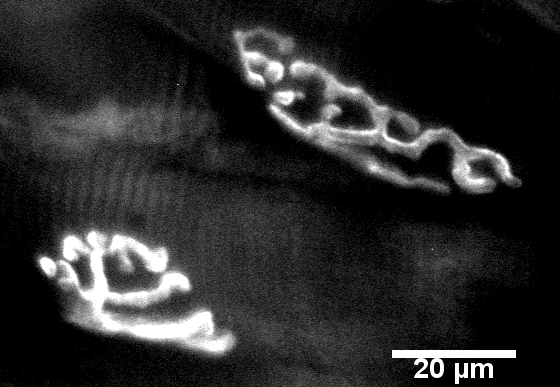


AChR

NF + Syn

Merge

Control


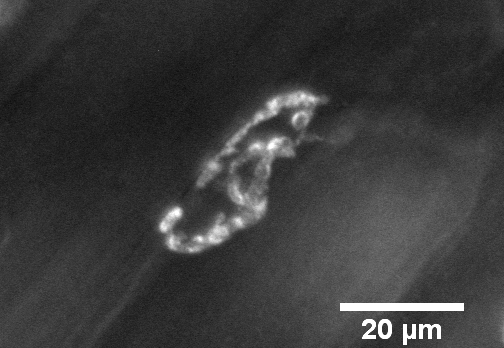

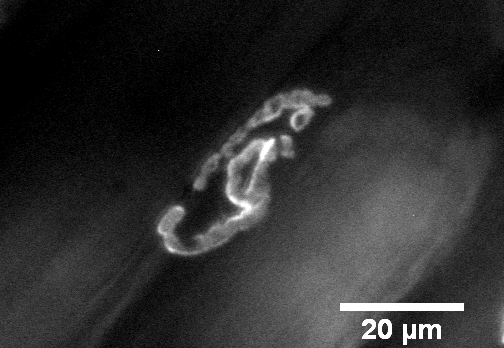

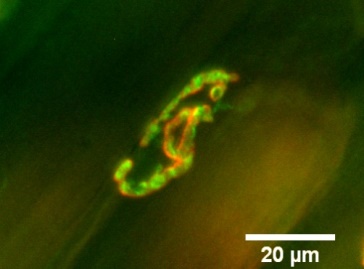


M-LAP1^-/-^


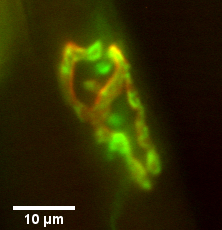

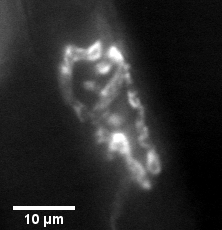

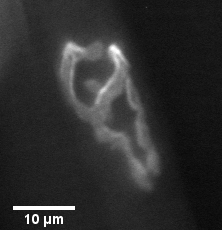

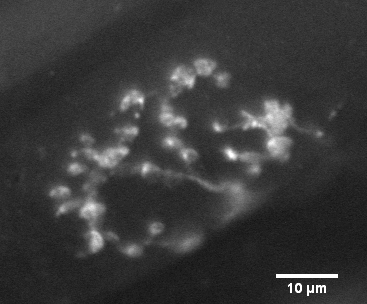

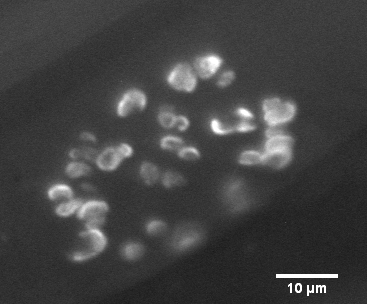

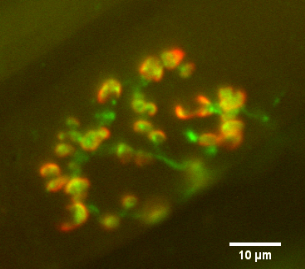


Control

M-LAP1^-/-^

Merge

NF + Syn

AChR

**B**

**Supplementary Figure 8 – Pre- and post-synaptic registration and neuronal sprouting in 12 week old mice. A** Motoneurons were stained with antibodies against neurofilament and synaptophysin (green; NF + Syn) and post-synaptic AChRs were stained with 594-α-BuTx (red; AChR). Good registration was observed as shown in the merged image. Scale bar =20μm

**B** Sprouting was also noted in both control and M-LAP1^-/-^ mice (indicated by red arrows). Scale bar =10μm

**Supplementary Table 2.** Example of neurophysiology data from the right *trapezius* of index patient. Muscle was subject to three trains comprising of 5 stimulations at 3Hz, and the area decrement of compound muscle action potential was recorded.

| Stimulation | Area decrement (%) |
| --- | --- |
| 5@3Hz | -26.5 |
| 5@3Hz | -18.0 |
| 5@3Hz | -18.6 |
| Mean area decrement | -21.0 |

**RNA-seq data**

**Supplementary Table 3.** RPKM (reads per kilobase per million) values from the RNA-seq analysis of 6 and 12 week old control and M‑LAP1^‑/‑^ mice (three mice per group, each column shows data from an individual mouse).

|  | 6 weeks old | | | | | | 12 weeks old | | | | | | |
| --- | --- | --- | --- | --- | --- | --- | --- | --- | --- | --- | --- | --- | --- |
| Gene | control | | | M-LAP1^-/-^ | | | control | | | M-LAP1^-/-^ | | | |
| *Chrna1* | 8.64 | 9.46 | 7.83 | 8.43 | 8.52 | 8.54 | 6.51 | 6.83 | 7.54 | 19.46 | 23.64 | 18.71 |  |
| *Chrnb1* | 29.95 | 32.98 | 33.14 | 28.89 | 33.31 | 32.75 | 28.85 | 21.29 | 24.64 | 39.99 | 43.31 | 33.93 |  |
| *Chrnd* | 1.72 | 2.19 | 2.95 | 1.97 | 1.76 | 1.75 | 3.19 | 3.44 | 3.74 | 5.76 | 6.62 | 7.07 |  |
| *Chrne* | 18.91 | 18.33 | 22.13 | 17.83 | 18.46 | 18.61 | 19.39 | 18.46 | 20.59 | 17.78 | 17.12 | 12.73 |  |
| *Chrng* | 0.00 | 0.00 | 0.00 | 0.00 | 0.00 | 0.00 | 0.23 | 0.49 | 0.31 | 13.99 | 20.03 | 20.17 |  |
| *Dok7* | 6.12 | 6.77 | 6.33 | 6.08 | 6.86 | 5.76 | 12.55 | 13.51 | 13.97 | 13.35 | 13.85 | 17.39 |  |
| *Rapsn* | 24.02 | 23.80 | 27.37 | 25.53 | 25.83 | 24.48 | 33.40 | 30.58 | 31.95 | 27.24 | 26.69 | 25.34 |  |
| *Musk* | 0.42 | 0.47 | 0.54 | 0.86 | 0.67 | 0.41 | 0.72 | 0.85 | 0.76 | 1.56 | 1.98 | 1.83 |  |
| *Utrn* | 2.58 | 3.38 | 2.75 | 2.62 | 3.13 | 3.01 | 2.69 | 2.77 | 2.83 | 2.66 | 2.35 | 3.03 |  |
| *Dmd* | 7.55 | 6.32 | 7.14 | 6.77 | 7.08 | 6.61 | 5.02 | 5.00 | 5.4 | 4.12 | 3.37 | 3.5 |  |

**Supplementary Table 4**. Analysis of neuromuscular junction ultrastructure from electron micrographs of 12 week old M‑LAP1^‑/‑^ and control mice

|  | Control | | M-LAP1^-/-^ | |
| --- | --- | --- | --- | --- |
| Feature measured | Mean | SD | Mean | SD |
| width of synaptic cleft (μm) | 0.068 | 0.013 | 0.059 | 0.015 |
| presynaptic length (μm) | 4.830 | 2.058 | 5.137 | 2.750 |
| postsynaptic length (μm) | 15.430 | 8.854 | 14.810 | 9.239 |
| Folding index* | 3.259 | 1.318 | 3.001 | 1.519 |
| pre-synaptic area (μm^2^) | 3.962 | 3.947 | 4.846 | 3.086 |
| post-synaptic area (μm^2^) | 2.805 | 1.613 | 2.674 | 1.755 |
| number of vesicles per pre-synaptic area | 27.200 | 24.900 | 21.140 | 16.360 |
| Number primary folds | 7.269 | 4.557 | 6.917 | 5.389 |
| Length of primary folds | 0.659 | 0.228 | 0.704 | 0.250 |
| Number secondary folds | 1.615 | 1.627 | 1.250 | 1.452 |

*Folding index = postsynaptic length/presynaptic length

*Number of NMJs measured = 26 (control) and 22 (M-LAP1^-/-^), from two mice per group.*

**Supplementary References**

Cossins J, Webster R, Maxwell S, Burke G, Vincent A, Beeson D. A mouse model of AChR deficiency syndrome with a phenotype reflecting the human condition. Hum Mol Genet 2004; 13(23): 2947-57.

Katz B, Thesleff S. On the factors which determine the amplitude of the miniature end-plate potential. J Physiol 1957; 137(2): 267-78.

Linder TM, Quastel DM. A voltage-clamp study of the permeability change induced by quanta of transmitter at the mouse end-plate. J Physiol 1978; 281: 535-58.

McLachlan EM, Martin AR. Non-linear summation of end-plate potentials in the frog and mouse. J Physiol 1981; 311: 307-24.
